# The role of macrophages in the mitigation by decitabine of acute allograft rejection

**DOI:** 10.3389/frtra.2025.1723396

**Published:** 2025-12-11

**Authors:** William N. Daccarett-Bojanini, Manuel Sollmann, Kristine M. Yarnoff, Nicola M. Heller, Jeffrey M. Dodd-o

**Affiliations:** 1 Department of Anesthesiology and Critical Care Medicine, Johns Hopkins Medical Institution, Baltimore, MD, United States; 2 Division of Anesthesiology and Critical Care Medicine, University of Leipzig Medical Center, Leipzig, Germany

**Keywords:** lung transplantation, acute rejection, macrophage, decitabine, immune tolerance

## Abstract

**Introduction:**

We have recently shown that the DNA hypomethylating agent decitabine (DAC) rescues lung allografts from acute rejection. This involves a mechanism that is dependent on host CD4+ FoxP3+ T cells for maximal benefit. DAC treatment also reduces host T-cell IFN-*γ* production. We therefore hypothesized that DAC may also reduce host macrophage activation. Our objective was to determine if an effect on macrophages contributes to the beneficial effects of DAC in transplantation.

**Methods:**

In murine orthotopic lung transplant, hosts were treated on post-op day 3-8 with Clodronate (*n* = 5), DAC (*n* = 9), or DMSO (*n* = 11).

**Results:**

Partial macrophage depletion (clodronate) improves allograft gross and histologic integrity. DAC-mediated allograft rescue was associated with reduced host macrophage recruitment into allograft airways, reduced activation of recruited macrophages, and regeneration of donor resident alveolar macrophages.

**Discussion:**

These findings suggest that infiltrating host macrophages promote allograft rejection. They also suggest that donor alveolar health is indicative and/or promoting of allograft tolerance.

## 1 Introduction

Though lung transplantation remains the final treatment option for many patients with end-stage lung disease, acute cellular rejection is experienced by nearly 1/3 of transplant recipients within the first year (1), and graft loss occurs in 46% of patients by 5 years (2). Current calcineurin inhibitor-based immune suppression regimens effectively block proliferation of injurious CD4 and CD8 cells, but do so at the expense of reducing immune-tolerizing regulatory T cells (3). This compromises their effectiveness against acute (4) and chronic (5) rejection. A more precision-based interruption of the immune cascade preserves the possibility that immune-tolerizing arms of the cascade can compensate. Intrinsic to the success of such an approach is correctly identifying both, which cascades are critical and which points within those cascades are rate-limiting. Thus, in a murine orthotopic lung transplant model, rejection persists in either the isolated absence of Tbet-mediated IFN-*γ* production (6) or the isolated neutralization of CD8 IL-17 by azithromycin (7). By contrast, CD154-mediated co-stimulatory blockade impairs allospecific CD4 and CD8 effector responses while enhancing Treg populations (8), preserving allograft viability for at least a year in this model (9).

The DNA methyltransferase 1 inhibitor decitabine (DAC) reduces lung rejection through activity against multiple arms of the immune response (10). DAC (vs. DMSO) treatment increases the percentage of CD4+ Tregs and CD8+ Tregs expressing GATA-3, decreases the secretion of IFN-*γ* from both CD4 and CD8 cells, and decreases secretion of both TNF-ɑ and IL-17 from CD8 cells (10, 11). While GATA-3 is a signature transcription factor for Th2T cells, inhibition of IFN-*γ* would be expected to reduce macrophage activation (12).

We hypothesized that, in addition to its described effect on inflammatory and regulatory T cells, host treatment with DAC would attenuate rejection by altering the phenotype of macrophages sequestered into the allograft. Allografts from hosts depleted of macrophages (liposomal clodronate) beginning post-op day (POD) 3 showed significantly reduced acute rejection, supporting a role for macrophages in the acute rejection process. Compared to vehicle-treated hosts, DAC treatment beginning POD 3 reduced post-transplant injury at POD 9 and lead to a change in the composition and phenotype of recruited macrophages in the lung.

## 2 Methods

### 2.1 Mice

Male and female C57BL/6 (H-2b, Strain #:000664), C57BL/6 CD45.1 (#033076), and BALB/c (H-2d, Strain #000651) mice (25–35 g) from Jackson Laboratory (Bar Harbor, ME, USA) were bred and housed in a pathogen-free facility before surgery. Open access conditions existed after surgery. All animal protocols were approved by the Johns Hopkins Animal Care and Use Committee (protocol: M024M180).

### 2.2 Lung transplant and pharmacologic administration

Donor left lungs (BALB/c mice) were transplanted into wild-type C57BL/6 or CD45.1 mice using cuffed techniques previously described (13). Intraperitoneal DAC (1 mg/kg) or vehicle (DMSO), from Sigma-Aldrich, St. Louis, MO, USA, was administered on days 3, 4, 5, and 8 post-operatively (10). In separate experiments, liposomal clodronate (200 mcl) or empty Encapsome (200 mcg, both from Encapsula Nanosciences, Brentwood, TN, USA) was administered via the intrajugular route on days 3, 5, and 7, and by intraperitoneal route on days 4, 6, and 8. The lungs were harvested on day 3 or 9 post implant. In some cases, the lungs from naïve mice were harvested.

### 2.3 Flow cytometry

The left and right lungs were enzymatically digested separately to generate a single-cell suspension as previously described (10). The cells were prepared for flow cytometry using fluorochrome-conjugated antibodies for surface and intracellular markers (Supplementary Figure S1). A fixable, UV-excitable Blue Dead Cell Stain (Invitrogen, Carlsbad, CA, USA) was used for live–dead discrimination, and UltraComp eBeads (eBioscience, San Diego, CA, USA) were utilized for compensation. Flow cytometry analysis was performed using a Cytek Aurora flow cytometer (Cytek, Fremont, CA, USA), and data were analyzed using FlowJo software (Tree Star Inc, San Carlos, CA, USA).

Flow cytometry gating was performed to identify and quantify specific immune cell populations in the allograft (Supplementary Figure S2). Events were first gated to exclude debris and doublets, selecting single cells (SSC-A vs. SSC-H). Live cells were identified using the viability dye and gated accordingly. Leukocytes were identified using CD45.1 (host) or CD45.2 (donor). B cells and T cells were gated based on CD 45R-B220 and CD3, respectively. Within the T-cell population, CD4 + and CD8+ T cells were selected separately. Markers for CD11c, CD11b, MHC II, Siglec F, Ly6G, Ly6C, CD64, and CD24 were used to identify macrophages, eosinophils, dendritic cells, and neutrophils as described previously (14). To determine the corresponding leukocyte markers, the cells were treated with antibodies against CD123, CD88, CD21, CD40, CD80, CD24, TGF-beta, IL-10, Ki-67, CD115, Ly6C, TNF-α, CD86, CD36, F4/80, DECT-1, CD192, MMR, MHC II, CD163, iNOS, and CD274. Mean fluorescence intensity (MFI) was defined as the geometric mean fluorescence intensity of the positive population.

### 2.4 Histopathology and acute rejection pathology scoring

Grafts were fixed in 4% formalin after harvesting. Embedding (paraffin), sectioning, and staining with Hematoxylin & Eosin were performed by the Reference Histology Core of Johns Hopkins University School of Medicine. Two blinded observers scored stained sections using standard criteria developed by the International Society for Heart and Lung Transplantation (ISHLT) grade A and B Lung Rejection Study Group (15).

### 2.5 Immunofluorescence staining

Quadruple immunolabeling for CD4 + CD8 + CK19 + F4/80 was performed at the Oncology Tissue Services Core of Johns Hopkins University School of Medicine. Immunostaining was performed sequentially using primary antibody, detection, using an anti-rabbit HQ detection system (Cat. #7017936001 and #7017812001, Roche Diagnostics, Indianapolis, IN), and signal amplification, with OPAL fluorophores (Akoya Biosciences, Marlborough, MA) diluted 1:200 in 1X Plus Amplification Diluent (Cat. #FP1498, Akoya Biosciences, Marlborough, MA). For CD8 detection, a rabbit anti-rat linker antibody (1:500; Cat. #AI4001, Vector Labs, Newark, CA) was used. Additional antibodies used were anti-CD4 (1:200; Cat. #ab133616, Abcam, Waltham, MA) detected with OPAL 570, anti-CD8 (1:125; Cat. #4SM16, Invitrogen, Waltham, MA) detected with OPAL 690, anti-CK19 (1:1,000; Cat. #ab133496, Abcam, Waltham, MA) detected with OPAL 520, and anti-F4/80 anti-F4/80 (1:200 dilution; catalog# 70076S, Cell Signaling Technology, Danvers, MA) detected with OPAL Polaris 780. Finally, sections were counterstained with spectral DAPI (Cat. #FP1490, Akoya Biosciences, Marlborough, MA) and mounted with Prolong Gold (Cat. #P36930, Thermo Fisher Scientific, Waltham, MA). Slides were viewed and scanned using the Olympus IX83 Inverted Microscope FISHscope and the Olympus CellSens software. Images were analyzed using ImageJ (see Supplementary for further details).

### 2.6 Statistical analysis

To assess the differential expression of markers on allograft live cells across different treatment groups (DMSO vs. DAC and Clodronate vs. Encapsome), a series of pairwise comparisons was performed.

Statistical comparisons between two groups were performed using a two-tailed Mann–Whitney (unpaired, non-parametric). Outliers, defined as below Q1 – 1.5×IQR for low-range outliers and above Q3 + 1.5×IQR for upper-range outliers, were excluded.

For evaluation of flow cytometry MFI data, markers reflecting similar pathways were grouped together and analyzed as a group. Thus, C5aR1 and CR2/CR1 reflect the complement system; B7-H1 reflects the PD-1 pathway; CD36 and Dectin 1 reflect scavenger-mediated pathways; CD40, CD80, and CD86 reflect co-stimulation; CD64 reflects antibody-mediated pathways; and iNOS and CD163 reflect general inflammatory markers. Values were compared with *T*-tests, with *P*-values adjusted for multiple testing using the adaptive Benjamini–Krieger–Yekutieli (BKY) false discovery rate (FDR) procedure with a Q value (FDR threshold) of 0.05. In other cases, comparisons were made using analysis of variance with Dunns.

### 2.7 Visualization

For visualization in the volcano plots, log2 fold change (log2FC) was calculated as the log2-transformed ratio of the percentage of allograft live cells expressing each marker between treatment groups. Separate volcano plots were generated using the MFI of each marker. Log2 fold change was computed as the ratio of MFI values between treatment conditions. The *P*-values for both analyses were obtained from the Mann–Whitney test and transformed using −log10(*p*-value) to represent statistical significance. Markers were classified as upregulated (log2FC > 0, bright blue) or downregulated (log2FC < 0, bright red). Significant markers (*p* < 0.05, −log10 *p*-value > 1.301) were shaded in green. Non-significant markers were shaded in gray. This approach allowed visualization of differential expression both in terms of the percentage of marker-positive cells and the intensity of marker expression on individual cells. All analyses and figure generation, including volcano plots, were performed using GraphPad Prism (GraphPad Software, San Diego, CA, USA; version 10.4.1).

## 3 Results

### 3.1 DAC treatment reduces interstitial cellular infiltration and preserves limited donor immune cell viability

**Figure 1:**
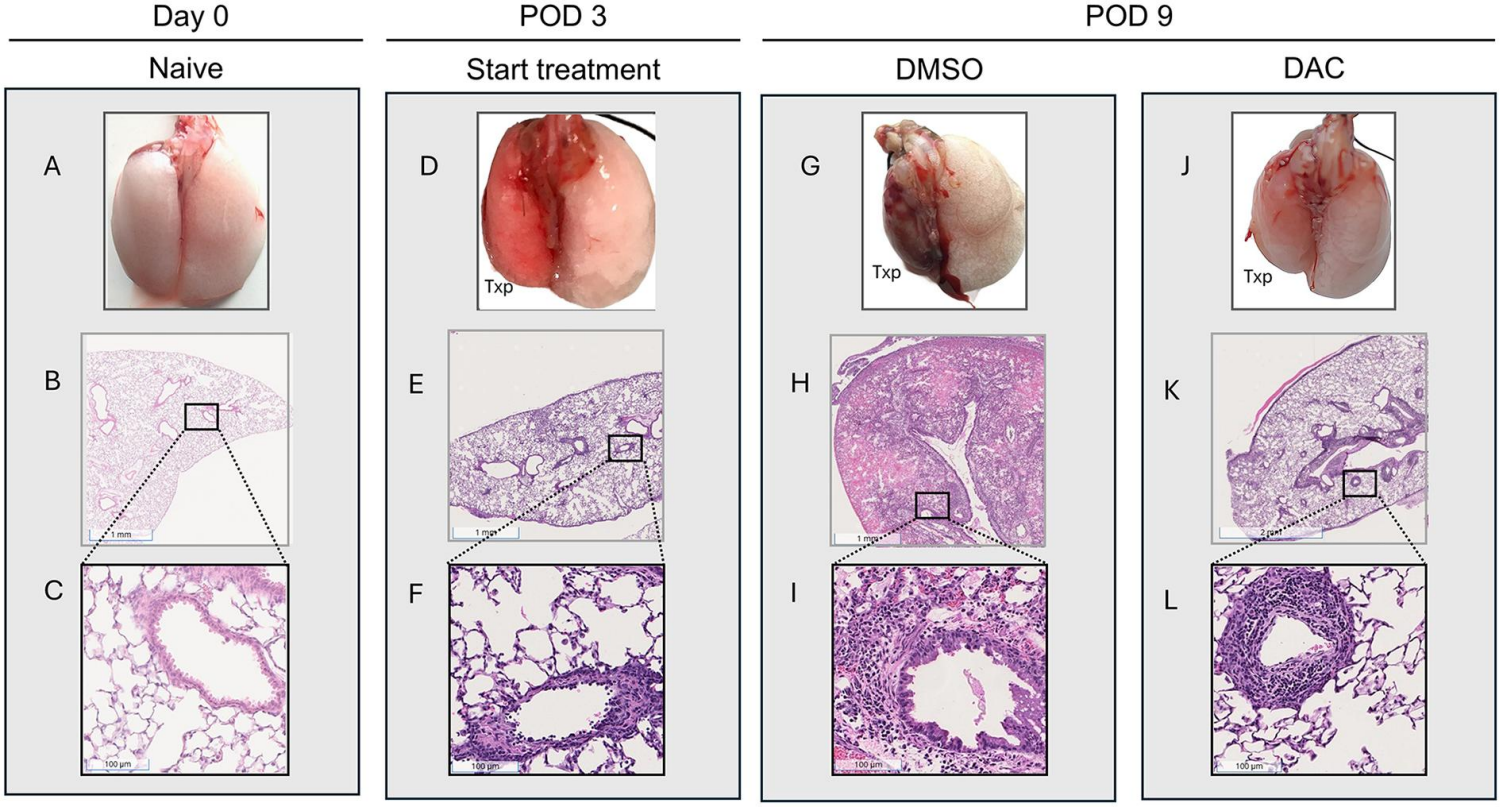
Decitabine attenuates lung allograft rejection. Gross morphology [**(A)**, **(D)**, **(G)**, and **(J)**] and histologic hematoxylin and eosin (H&E) staining [**(B,C)**, **(E,F)**,**(H,I)**, and **(K,L)**, 1× and 20× magnifications] of BALB/c naïve lungs and allografts harvested 3 or 9 days post transplantation into wild-type C57BL/6 hosts. Mice harvested 9 days were treated with either vehicle (DMSO, intraperitoneally) or decitabine (DAC, 1 mg/kg, intraperitoneally) on post-transplant days 3, 4, 5, and 8. DAC treatment preserved lung architecture and reduced inflammatory cell infiltration compared to DMSO-treated controls. TPX: Left Lung Allograft, L: Left, and R: Right*.*

This model of lung transplantation led to gross lung consolidation and diffuse dense cellular infiltration histologically (DMSO, POD 9). Cellular infiltration was already present at the time of treatment initiation (POD 3 vs. POD 0) (Figures 1E,F vs. B,C). Infiltration continued at a significantly reduced rate in DAC-treated hosts vs. DMSO-treated hosts (POD 9, DMSO vs. DAC) (Figures 1K,L vs. H,I). Thus, perivascular cellularity is somewhat less prominent, and interstitial as well as airway cellularity is nearly absent, in POD 9 allografts from DAC- (vs. DMSO-) treated hosts. ISHLT scoring confirmed the progression of rejection in allografts of DMSO- vs. DAC-treated hosts (Supplementary Figure S3).

**Figure 2:**
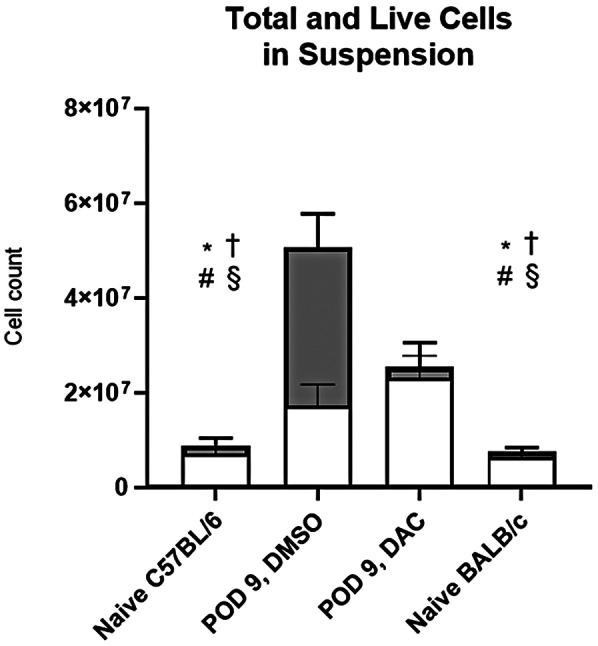
Total donor and host cells comprising live cell suspensions. Histograms depicting the total single-cell suspension counts (black histogram) and absolute number of live cells (white histogram). *Total cells *P* < 0.05 vs. DMSO; † Total cells *P* < 0.05 vs. DAC; # Live cells *P* < 0.05 vs. DMSO, § Total cells *P* < 0.05 vs. DAC. Data are presented as mean ± SEM (*N* = 12 naïve C57BL/6, 9 DMSO, 11 DAC, 10 naïve BALB/c).

The effect of host DAC therapy on allograft cell viability was determined both in-total and with respect to donor- vs. host-cell contribution. Single-cell suspensions from allografts were analyzed using live/dead staining and surface markers for CD45.2 and CD45.1 (Figure 2). Flow cytometry confirmed that, compared to naïve lungs, allografts from both DMSO-treated and DAC-treated hosts each have more total cells and more live cells. In allografts from POD 9 DAC-treated hosts, the percentage of live cells is similar to that of naïve lungs (naïve host vs. DAC, 82% ± 3% vs. 91% ± 1%, *p* NS). By contrast, in allografts from POD 9 DAC-treated hosts, the percentage of live cells was significantly smaller (naïve host vs. DMSO, 78% ± 4% vs. 34 ± 11, *P* = 0.04).

Of the total live cell suspensions from naïve host lungs and allograft from either host-treatment group, host immune cells comprise 85%–90% of all live allograft (Supplementary Figure S4A). In allografts, the percentage of live cells of donor origin was 0.2 ± 0.1 in DMSO-treated hosts vs. 5.2 ± 1.1 in DAC-treated hosts (*P* = 0.07) (Supplementary Figure S4b). Thus, host cells infiltrate allografts regardless of host treatment, but DAC treatment of hosts tends to preserve the viability of allograft cells in general and of donor-derived immune cells in particular.

### 3.2 DAC treatment preserves an allograft live immune cell-type composition similar to that of naïve host lungs

**Figure 3:**
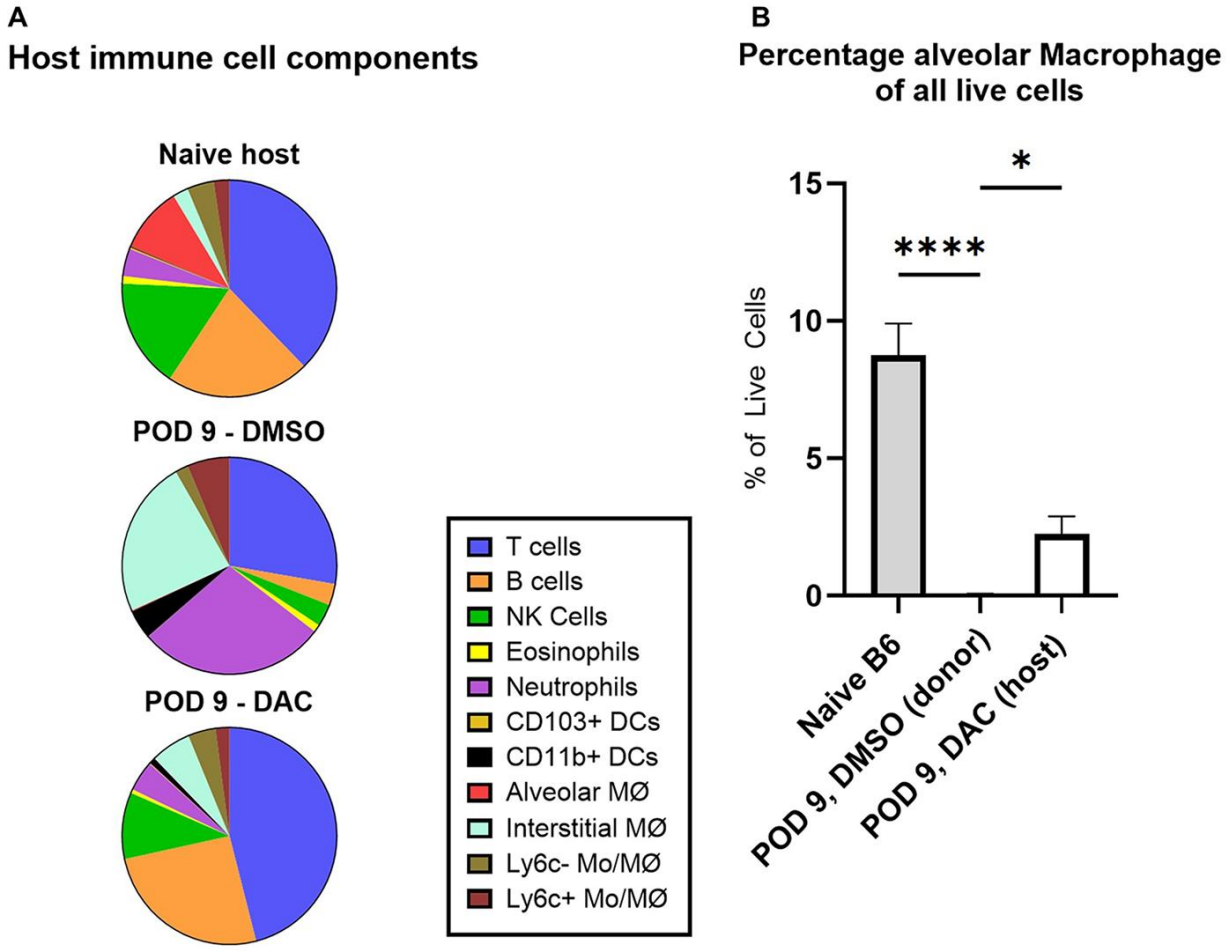
Component host and donor cells comprising live cell suspensions. **(A)** Pie chart showing live host immune cell components as percentage of total live cell suspension in naïve host, POD 9 DMSO allograft, and POD 9 DAC allograft. **(B)** Histograms depicting live alveolar macrophages (CD45 + B220-CD3-NK1.1-Siglec F+) as a percentage of total live cells in naïve donor lungs, POD 9 DMSO allografts, and POD 9 DAC allografts. POD 9 DMSO allografts had essentially no host alveolar macrophages. POD 9 DAC alveolar macrophages are of donor origin, as there were essentially no host-origin alveolar macrophages in these allografts. Data are presented as mean ± SEM (*N* = 12 naïve host, 9 DMSO, 11 DAC). *P* ≤ 0.05 (*), *P* ≤ 0.01, and *P* ≤ 0.0001 (****).

Having observed that a small number of CD45.2 cells in allografts from DAC-treated hosts were donor immune cells in any POD 9 allograft, we next focused on comparing the total immune cell composition within naïve hosts, POD 9 DMSO, and POD 9 DAC allografts (Figure 3A). Naïve lungs were similar, with the exception of slightly more neutrophils and fewer alveolar macrophages (CD45 +  B220-CD3-NK1.1-Siglec F+) in the CD45.1 (naïve host) lungs. Compared to CD45.2 (naïve donor) lungs, allografts from POD 9 DMSO-treated hosts are essentially depleted of alveolar macrophages and NK cells and demonstrate a quantitative increase in interstitial macrophages and neutrophils. By contrast, the allograft live CD45.1 population in allografts from POD 9 DAC-treated hosts mirrored that of the naïve CD45.1 mouse, with the exception of a lack of alveolar macrophages. Instead, CD45.2 cell population resident alveolar cells (CD45 +  B220-CD3-NK1.1-Siglec F+) comprise a percentage of total live allograft cells of POD 9 DAC-treated hosts that is similar to that of resident alveolar macrophages in naïve donor lungs (naïve vs. POD 9 DAC, 8.7 ± 1% vs. 3.1 ± 1%, *P* = 0.07) (Figure 3B and Supplementary Figure S5). Thus, DAC treatment of hosts preserves an immune cell composition in allografts that is similar to that of a naïve host, with alveolar cells being of donor origin but other immune cells being of host origin.

### 3.3 DAC treatment restricts donor macrophage infiltration into allograft alveoli and colocalization with T cells

**Figure 4:**
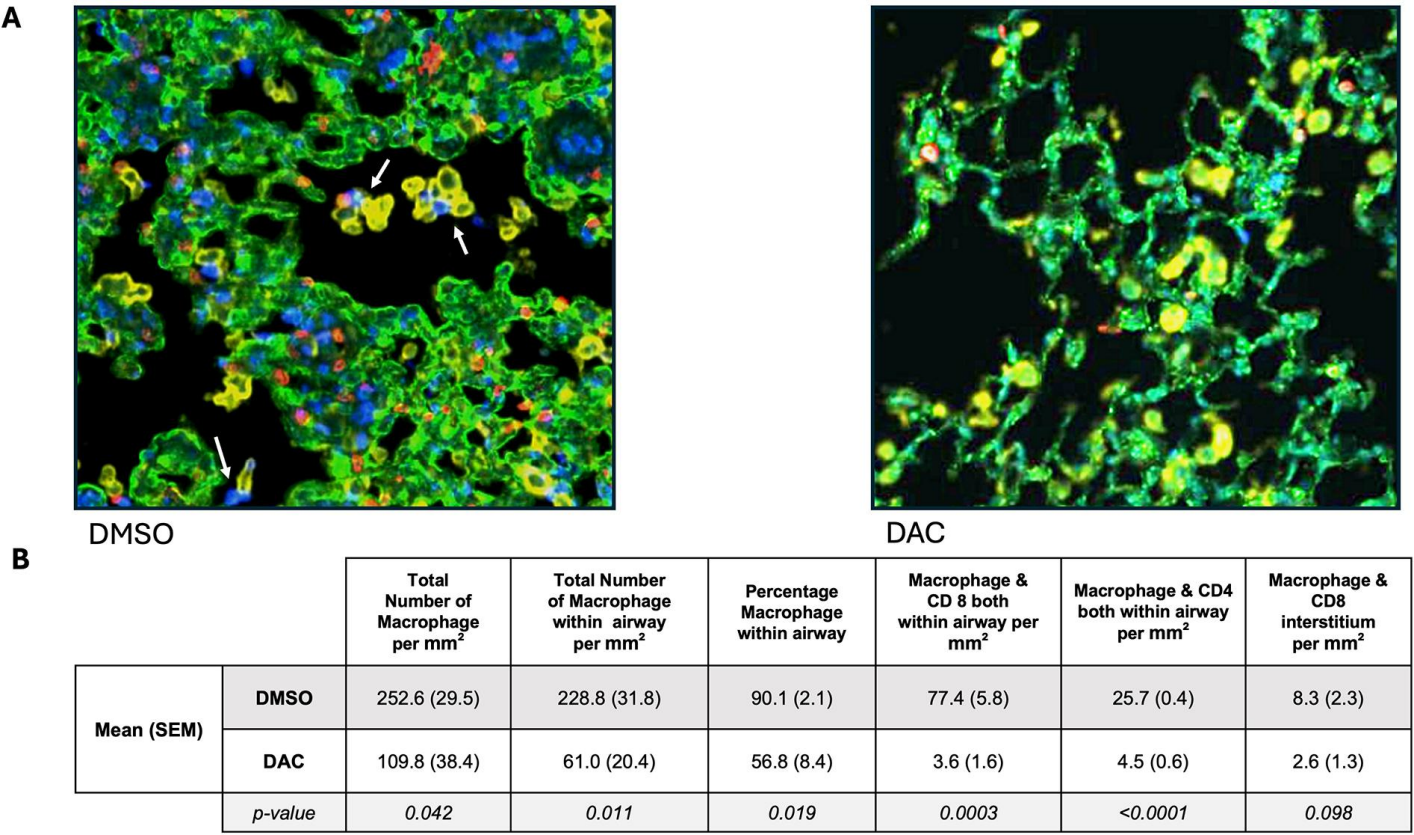
DAC treatment restricts cellular infiltration to the extra-alveolar spaces and decreases macrophage–T-cell colocalization. **(A)** Representative immunofluorescence images of allografts harvested 9 days post-transplant from DMSO- and DAC-treated hosts. Sections were stained for CK-19 (green), F4/80 (yellow), CD4 (red), and CD8 (blue) to assess macrophage T-cell distribution across different lung regions. DAPI (cyan) marks nuclear staining. Arrow indicates site of colocalization of macrophage with CD4 or CD8T lymphocyte. **(B)** Tabular representation of DAC effect on macrophage distribution and macrophage: T-cell colocalization. Scale bars = 200 μm*.*

We next compared the effect of DAC on macrophage distribution and colocalization with T cells within allografts. Immunofluorescence analysis for macrophage and T-cell markers (F4/80, CD4, and CD8) and the epithelial marker CK-19 was performed (Figure 4) showing widespread distribution of macrophages throughout all regions of the lung allografts in POD 9 DMSO-treated hosts. Compared to POD 9 DMSO allografts, allografts from POD 9 DAC-treated hosts show significantly fewer macrophages per mm^2^ overall (DMSO vs. DAC 252.6 ± 29.5 vs. 109.8 ± 38.4, *P* = 0.04), and significantly fewer macrophages per mm^2^ within the airway (DMSO vs. DAC, 228.8 ± 31.8 vs. 61 ± 20.4, *P* = 0.01). Intra-alveolar colocalization of macrophages with CD8+ (DMSO vs. DAC, 77.4 ± 5.8 vs. 3.6 ± 1.6, *P* = 0.0003) and CD4+ (DMSO vs. DAC, 25.7 ± 0.4 vs. 4.5 ± 0.6, *P* < 0.0001) cells per mm^2^ is also less common in allografts from POD 9 DAC-treated hosts, although it can be observed in these allografts as well. Within the interstitium, macrophage colocalization with CD8 + cells (DMSO vs. DAC, 8.3 ± 2.3 vs. 2.6 ± 1.3 *P* = 0.098) and with CD4 + per mm^2^ occurs equally rarely regardless of treatment group. Thus, DAC treatment is associated with a reduced density of macrophages within the lung, a lower percentage of those macrophages being within the airway, as well as a decreased colocalization of macrophages with CD4 and CD8 cells.

### 3.4 Preserved macrophage viability is required to maximize acute rejection

**Figure 5:**
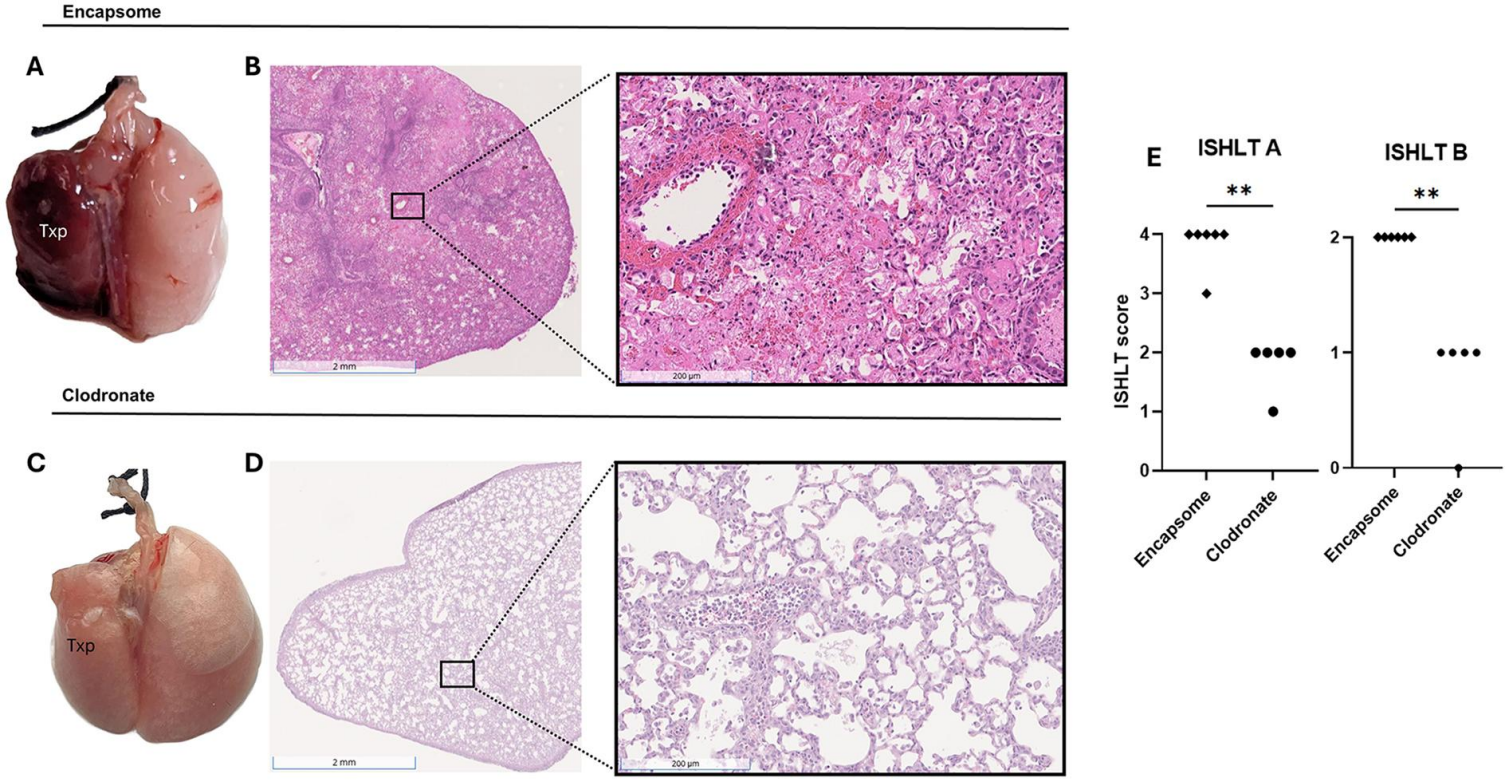
Preserved macrophage viability is required to maximize acute rejection gross morphology [**(A)** and **(C)**] and histologic H&E staining [**(B)** and **(D)**, 1× and 20× magnifications] of BALB/c lung allografts harvested 9 days post transplantation from hosts receiving Encapsome or Clodronate. ISHLT A and ISHLT B scoring is also displayed **(E)**. Mice were treated with either vehicle (Encapsome) or Clodronate (200 mcl) i.v. on post-transplant days 3, 5, and 7 and i.p. on post-transplant days 4, 6, and 8. TPX: Left Lung Allograft, L: Left, and R: Right.

Given the observed association of decreased rejection intensity with decreased macrophage recruitment into allografts of POD 9 DAC-treated hosts, we evaluated whether a clodronate-mediated reduction in allograft macrophages would reduce the magnitude of allograft injury in Encapsome-treated mice. Clodronate administration reduced the percentage of interstitial macrophages (Encapsome vs. clodronate, 20.1 ± 2 vs. 12.9 ± 1, *P* = 0.02) and increased the percentage of alveolar macrophages (Encapsome vs. clodronate, 0.01 ± 0.01 vs. 0.08 ± 0.05, *P* = 0.002) comprising the total live cell population (Supplementary Figure S6). Reducing allograft macrophage recruitment with systemic clodronate administration reduced gross (Figure 5A) and histologic (Figure 5B) allograft injury vs. allografts from Encapsome-treated mice (Figures 5C–E).

### 3.5 DAC treatment influences time course of host monocyte-derived macrophage recruitment into allograft

**Figure 6:**
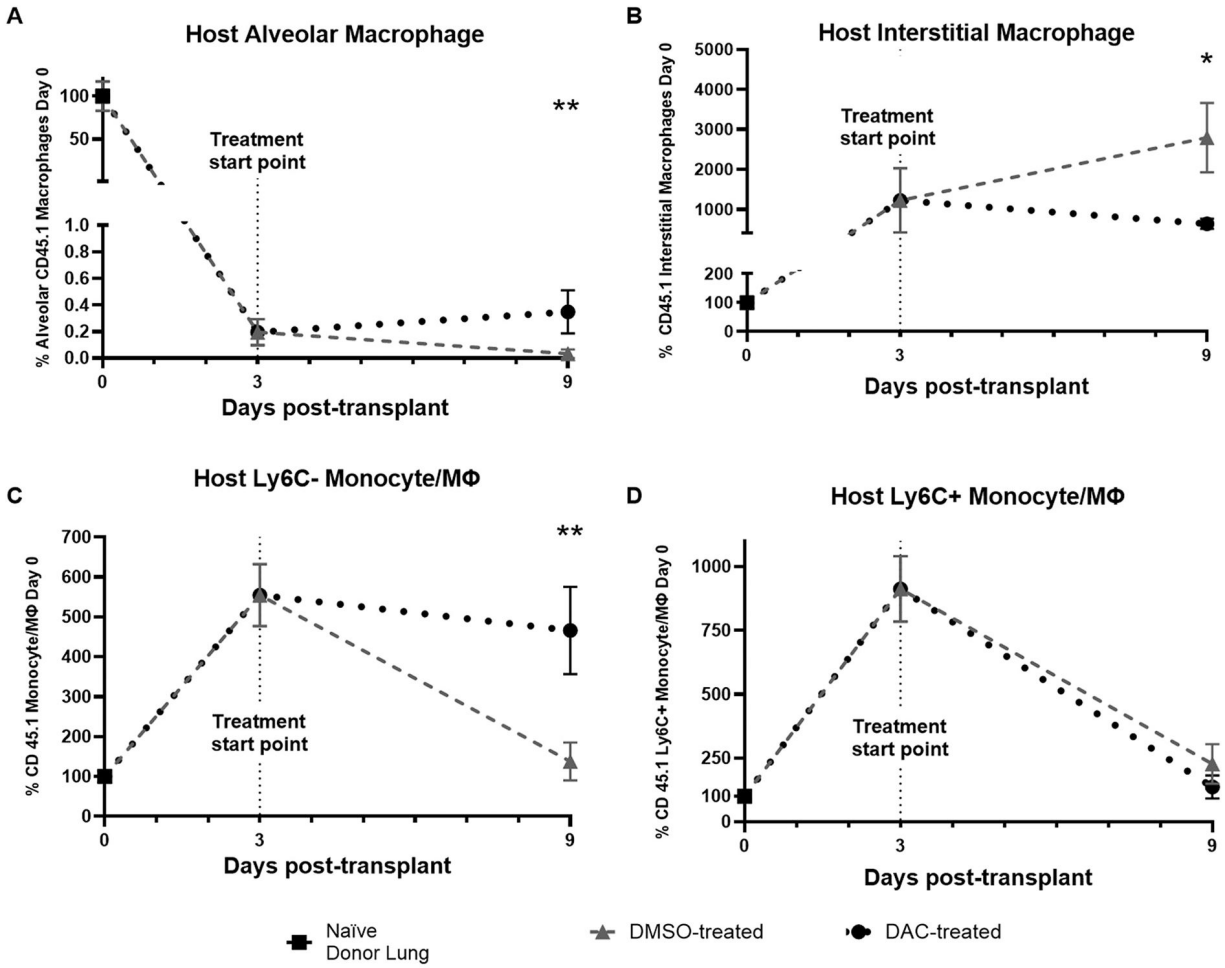
Time course of host macrophage infiltration into allograft. Plots displaying change in quantity (depicted as percentage of day 0 quantity) of CD45.1: **(A)** Alveolar macrophages; **(B)** interstitial macrophages; **(C)** Ly6C− monocyte-derived macrophages; and **(D)** Ly6C+ monocyte-derived macrophages at time of implant (Day 0), initiation of treatment (Day 3), and time of harvest (Day 9). Data are presented as mean ± SEM, with individual data points overlaid. *P* < 0.05 (*), 0.05 < *P* < 0.01 (**). (*N* = 12 naïve host, 4 mice at post-transplant day 3, 9 DMSO day 9, 11 DAC day 9).

We next explored the effect of DAC on the quantity of host macrophage populations within the allograft, expressing the quantity of live host macrophage cell types as a percentage of the number observed in a naïve host lung. Treatment began POD 3. By this point, an influx of host cells increased the number of host interstitial macrophages by 10-fold (baseline 100% to 1,000% at POD3) (Figure 6B), the host Ly6C− monocyte-derived macrophages population 5.5-fold (Figure 6C), and the host Ly6C+ monocyte-derived macrophages by approximately eight fold (Figure 6D). Between POD 3 and POD 9 in allografts from DMSO-treated hosts, the numbers of live host-derived monocyte-derived macrophages begin to decrease to near baseline levels, while the number of host-derived interstitial macrophages continues to rise. Between POD 3 and POD 9 in allografts from DAC-treated hosts, by contrast, the numbers of host-derived live Ly6C+ monocyte-derived macrophages also drop, but the numbers of live host-derived Ly6C− monocyte-derived macophages and interstitial macrophages plateau near POD 3 levels. Thus, when viewed as a percentage of the number of such cells in naïve mice, the surviving host-derived macrophage population in POD 9 DMSO (vs. DAC) allografts has a significantly larger increase in interstitial macrophage population (DMSO vs. DAC = 2,795% ± 868% baseline levels vs. 639% ± 127% naïve lung levels, *P* = 0.01), and a significantly smaller Ly6C− monocyte/macrophage population (DMSO vs. DAC, 138% ± 48% vs. 466% ± 109% naïve lung levels, *P* = 0.01). There is an extremely small population of resident alveolar macrophages of host origin in the allografts (Figure 6A). Thus, at the time of initiating treatment, allografts have received a massive influx of various host macrophage populations but have generated essentially no alveolar macrophages of host etiology. DAC treatment of hosts predominantly influences the continued viability of influxing host interstitial macrophages and Ly6C− monocyte-derived macrophages.

### 3.6 Host treatment with DAC changes the pattern of surface markers expressed by recruited host macrophages in allografts

**Figure 7:**
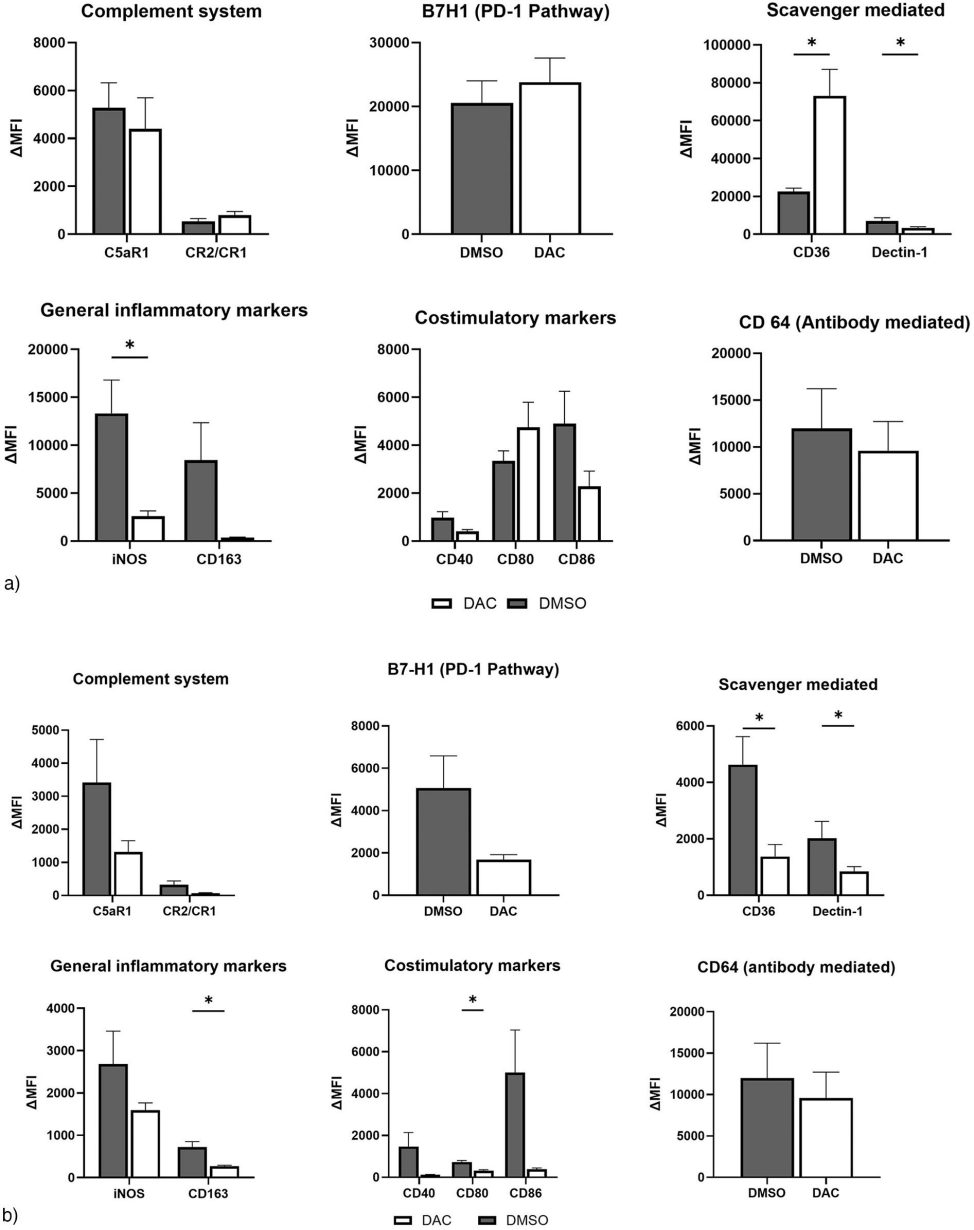
Effect of DAC on the phenotype of host interstitial macrophages and Ly6C− monocyte-derived macrophages. Histograms demonstrating the effect of DAC vs. DMSO treatment on the percentage of allograft **(a)** live host interstitial macrophages and **(b)** live host monocyte-derived macrophages expressing various markers. Markers reflecting different pathways were grouped together and analyzed as such. Data are presented as mean ± SEM. Values were compared with *T*-tests, with *P*-values adjusted for multiple testing using the adaptive BKY FDR procedure with a Q value (FDR threshold) of 0.05 (*N* = 9 DMSO, 11 DAC9).

We then explored the effect of host treatment with DAC on some of the various pathways by which recruited host macrophages may influence allograft tolerance. Our flow cytometry evaluation of inflammatory markers on live host-origin interstitial macrophages infiltrating POD 9 allografts suggests that, compared to DMSO treatment, DAC treatment of hosts increases CD36, decreases iNOS and Dectin-1, and trends to (*p* < 0.06) and CD163. On balance, this tends to suggest a more tolerogenic phenotype (Figure 7A, Supplementary Figure S7A). Similarly, our flow cytometry evaluation of inflammatory markers on live host-origin Ly6C− monocyte-derived macophages infiltrating POD 9 allografts suggests that, compared to DMSO treatment, DAC treatment of hosts decreases CD80 and CD64. This too, on balance, suggests that Ly6C− monocyte-derived macophages infiltrating allografts of DAC-treated (vs. DMSO-treated) hosts possess a less inflammatory phenotype (Figure 7B, Supplementary Figure S7b).

### 3.7 Host treatment with DAC promotes recovery of donor resident alveolar macrophage population

**Figure 8:**
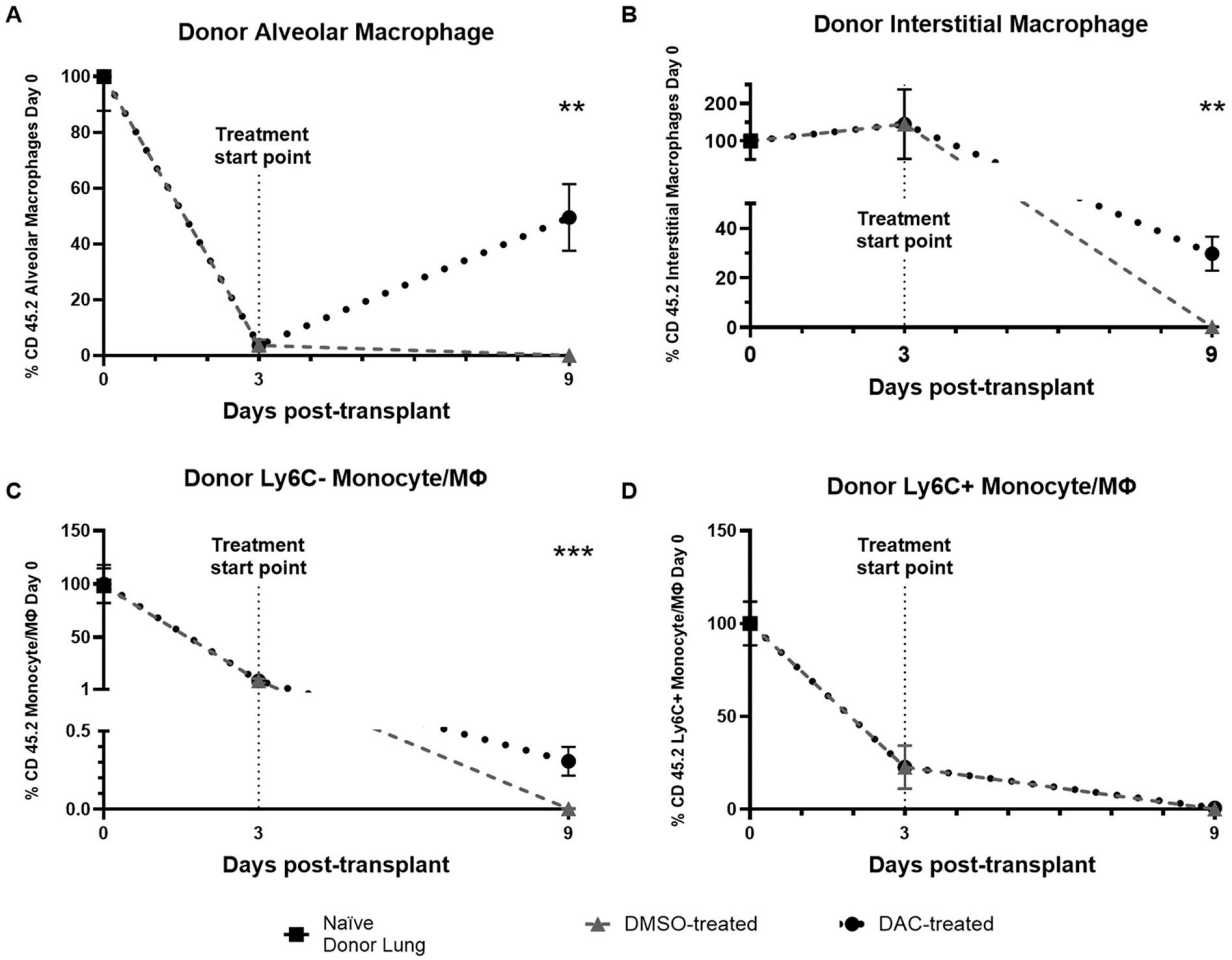
Time course of donor macrophage fluctuations within allografts. Plots displaying change in quantity (depicted as percentage of day 0 quantity) of CD45.2: **(A)** Alveolar macrophages; **(B)** interstitial macrophages; **(C)** Ly6C− monocyte-derived macrophages; and **(D)** Ly6C + monocyte-derived macrophages at time of implant (Day 0), initiation of treatment (Day 3), and time of harvest (Day 9). Data are presented as mean ± SEM, with individual data points overlaid. *P* ≤ 0.01 (**), *P* < 0.005 (***). *N* = 10 naïve donor, 4 day 3, 9 DMSO day 9, 11 DAC day 9.

We next explored the effect of DAC on the ability of allografts to preserve their host macrophage populations. We again present macrophage quantities as a percentage of the number seen in naïve donor lungs. Focusing on the change in live host macrophage population within grafts during the first 3 days of implantation with no host immunosuppression, we find a near complete loss of donor resident alveolar macrophages (Figure 8A) and donor Ly6C− monocyte-derived macophages (Figure 8B), a 75% drop in donor Ly6C+ monocyte-derived macrophages (Figure 8C), but a preservation of live donor interstitial macrophages. Between POD 3 and POD 9, essentially all live donor macrophage populations are lost in allografts from DMSO-treated hosts. By contrast, between POD 3 and POD 9 in allografts from DAC-treated hosts, approximately 30% of donor interstitial macrophages persist and the donor alveolar macrophage population recovers to approximately 40% of naïve lung values (Figure 8D). By POD 9, when evaluated as a percentage of the quantity present in naïve mice, DAC (compared to DMSO) allografts have more donor interstitial macrophages (DMSO vs. DAC, 0.24 ± 0.15 vs. 30 ± 7, % of naïve mice number *P* = 0.001) and donor alveolar macrophages (DMSO vs. DAC, 0 ± 0 vs. 40 ± 11, % of naïve mice number *P* = 0.003) (Figure 8A). Thus, DAC treatment of hosts appears to promote the recovery of the donor resident alveolar macrophage population and a partial preservation of donor interstitial macrophages in allografts.

### 3.8 Donor resident alveolar macrophage and interstitial monocyte-derived macrophage surface marker profile differs between allografts of DAC-treated hosts and naïve donor lungs

**Figure 9:**
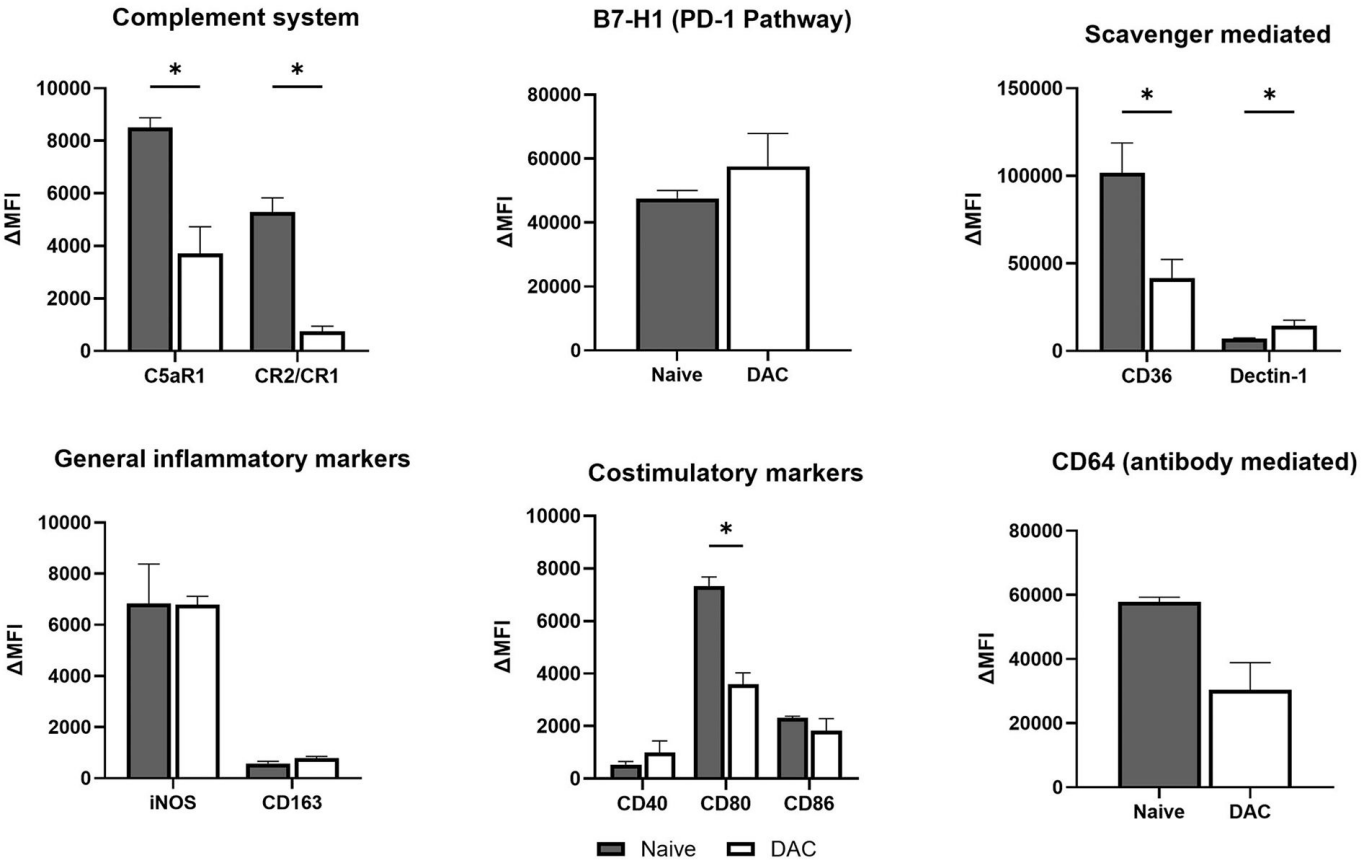
Effect of DAC on the phenotype of donor alveolar macrophages. Histograms demonstrating the effect of DAC vs. naïve lung on the percentage of allograft live donor alveolar macrophages expressing various markers. Data are presented as mean ± SEM. Values were compared with T-tests, with *P*-values adjusted for multiple testing using the adaptive BKY FDR procedure with a Q value (FDR threshold) of 0.05 (*N* = 10 naïve, 11 DAC). *P ≤ 0.05.

Using flow cytometry markers, we then explored the effect of host treatment with DAC on some of the various pathways by which residual donor macrophages may influence allograft tolerance. Compared to cells from naïve donor lungs, donor alveolar macrophages in harvested allografts from POD 9 DAC-treated hosts demonstrate increased C5aR1 and CR2/CR1, increased CD36 but decreased Dectin-1, and increased CD80 (Figure 9, Supplementary Figure S8). This represents a relatively activated cell type from many regards.

Using flow cytometry, we compared the percentage of live donor-origin CD45.2 interstitial macrophages in POD 9 allograft of DAC-treated hosts (vs. naïve donor lungs) expressing various markers of immune activity. DAC treatment increases B7-H1, iNOS, and Dectin-1 but decreases CD36. There is also a trend toward decreased expression of CD163. (Supplementary Figure S9a,S9b, with 9a being histograms). This suggests a complex effect of DAC on the inflammatory activation of residual donor host macrophages in the allograft.

### 3.9 DAC treatment reduces host neutrophil recruitment but increases host CD103 DC recruitment into allograft

Because macrophages may interact with neutrophils and dendritic cells, we evaluated the effect of host treatment with DAC on allograft neutrophil and CD103 + DC cell quantities. Compared to naïve lungs, the percentage of live CD45.1 cells comprising host neutrophils were more plentiful (naïve vs. DMSO 4.1 ± 0.5 vs. 21.7 ± 4, *P* = 0.0001) while the percentage of live CD45.1 cells comprising host CD103 + DCs were less abundant in POD 9 allografts from DMSO-treated hosts (naïve vs. DMSO 0.18 ± 0.05 vs. 0.01 ± 00, *P* = 0.0001) (Supplementary Figure S10). In POD 9 allografts from DAC-treated hosts, host neutrophils (naïve vs. DAC 4.1 ± 0.4 vs. 22.0 ± 4, *P* = 0.0001) were of similar quantity to those of naïve lungs and host CD103+ DCs (naïve vs. DAC 0.18 ± 04 vs. 0.05 ± 0.02, *P* = 0.06) were of similar quantity to (but trended lower than) those of naïve lungs. Thus, DAC treatment prevents the rise in host neutrophils, and the drop in host CD103 + DCs, that is seen in allografts from DMSO-treated hosts.

## 4 Discussion

### 4.1 Overview

Allografts from clodronate-treated hosts demonstrate a 40% reduction in host allograft macrophage population and a significantly reduced acute cellular rejection at POD 9. We demonstrate that DAC, initiated 72 h following graft implantation, attenuates murine lung allograft rejection seen at POD 9 through a process mediated, at least in part, through an effect on macrophages. The attenuated rejection is accomplished through a process that: (1) leads to a reduction in recruited host macrophages into the lung airways; (2) promotes the recovery of the donor resident alveolar macrophage population; (3) transitions the host and donor macrophages within the allograft to a less inflammatory phenotype; and (4) reduces the colocalization of host-derived macrophages with CD4 and CD8 cells within the alveoli.

### 4.2 DAC effects on host macrophage recruitment and phenotype

The quantity of host-derived interstitial macrophages remaining in harvested allografts from DAC-treated hosts, though reduced compared to that in allografts from DMSO-treated hosts, remains significant. Their phenotype appears to be one of reduced activation. Compared to interstitial macrophages of a naïve donor lung, an increased percentage of residual host interstitial macrophages of POD 9 allografts from DAC-treated hosts express B7-H1 and/or iNOS. Furthermore, B7-H1 is ligand for PD-1, and the binding of PD-1 by ligand is associated with prolonged transplant survival in a murine heterotopic heart transplant model (16). Additionally, blockade of PD-L1:PD1 interactions increases T-cell infiltration into the interstitium in a murine heart transplant model (17). In a heart transplant model whose strain combinations result in either chronic (BM12 deficient mice on B6 background −> B6 wild type) or acute (C3H −> BALB/c and B6−>BALB/c) rejection, high local macrophage iNOS levels promote rejection in a chronic rejection heart transplant model (18) but promote tolerance in an acute rejection model (19).

Compared to those from DMSO-treated hosts, macrophages from allografts of DAC-treated hosts are less likely to express iNOS or Dectin-1. High iNOS expression indicates a strong pro-inflammatory, potentially tissue-damaging macrophage state. In heart transplantation, local macrophage iNOS accelerates transplant rejection by a mechanism that is limited by stromal cell-derived IL-33 (18). Dectin-1 is a pattern recognition receptor that responds to DAMPs, and its genetic deletion on graft infiltrating macrophages leads to graft tolerance (20).

Host-derived Ly6C− monocyte-derived macrophages harvested from allografts of DAC- (vs. DMSO-) treated hosts less commonly express the co-stimulatory surface marker CD80. This suggests that these macrophages are less likely to be functioning as antigen presenting cells (21). They are also less likely to express Dectin-1*.* CD36 in macrophage membranes helps them recognize apoptotic cells. Phagocytosis of these cells (aka efferocytosis) leads to the colocalization of CD36 with platelet-activating factor receptor in the membrane, as well as with flotillin-1 (a constitutive lipid raft protein), and leads to an anti-inflammatory cytokine production pattern (IL10 ^high^/IL-12p40^low^) (22).

### 4.3 DAC effects on alveolar macrophage populations

Donor macrophage quantities measured progressively from time of transplant, through treatment initiation, to time of harvest suggests the preservation of small populations of donor alveolar macrophages and interstitial macrophages only in allografts from DAC-treated hosts. The donor alveolar macrophage population in POD 9 DAC allografts represents a numerical growth following initiation of DAC. Their surface marker profiles indicate they originate from local self-proliferation rather than migration from the interstitium. Resident alveolar macrophages, suppressive in nature, are felt to sense, respond to, and be affected by pro-inflammatory stimuli (23). Their capacity for self-replication, temporally associated with the resolution phase of the inflammatory stage of a viral process, may be linked to downregulation of the Wnt-β-catenin pathway (24).

We find that these donor resident alveolar macrophages in POD 9 DAC allografts have markers suggesting reduced stimulation of the complement cascade. In lung inflammatory states, complement system stimulation is associated with an activated alveolar macrophage phenotype. In a murine allergic asthma model of inflammation, fluid lavaged from the alveolar space demonstrated decreased neutrophilic and eosinophilic granulocyte accumulation when mice receive anti-C5aR1 treatment (25). In transplant rejection, differentially expressed genes common to both the complement activation cascade and to an alveolar macrophage transcription factor may provide the link between complement activation and rejection (26, 27).

Flow cytometry suggests that the phenotype of the donor alveolar macrophages harvested POD 9 from DAC-treated hosts is, interestingly, relatively activated compared to those from naïve donors. A greater percentage of such cells from POD 9 DAC-treated hosts express Dectin-1 and MMR, and a lower percentage express CD36 compared to when implanted (i.e., naïve lungs). In a skin transplant model, the presence of MMR is a requirement for rejection (28). Finally, CD36 helps meditate the recognition and phagocytosis of apoptotic cells by macrophages (a.k.a efferocytosis) (22). This process is important for graft tolerance (29).

Our immunofluorescence data suggest that, compared to those of POD 9 DAC lungs, the alveoli of POD 9 DMSO allografts contain more macrophages. Our flow cytometric data suggest the intra-alveolar macrophages from allografts of DAC- and DMSO-treated hosts have embryologically distinct origins. Resident alveolar macrophages are identified by the marker CD45 + B220-CD3-NK1.1-Siglec F +  (14) and derive embryologically from the yolk sac or fetal liver rather than from transformation of other cell types (30, 31). There is a growing awareness of their capacity for local regeneration, rather than any dependence upon migration (32). DAC treatment of hosts also results in a trend toward less frequent colocalization of intra-alveolar macrophages with CD4 + and/or CD8 + cells, and a trend toward less frequent colocalization of interstitial macrophages with CD8 + cells. We find that host treatment does not predict the likelihood that host interstitial macrophages will express MMR. Together, this suggests that the protective effect of DAC treatment is distinct from the MMR receptor-dependent, noncytotoxic CD4T cell-dependent, cytotoxic CD8+ T-cell-independent process of macrophage activation leading to the killing of allogeneic cells in a skin transplant model (33, 34).

### 4.4 Complex nature of DAC effects

The effect of DAC on macrophage activity is complex. Allografts harvested from DAC-treated hosts (compared to those from DMSO-treated hosts) contain host-derived interstitial macrophages that are more likely to express CD36 and Ly6C− monocyte-derived macrophages that are less likely to express CD163. CD163 expression characterizes macrophages involved in resolving inflammation and promoting tissue repair through the release of IL-10 (35).

The capacity to promote donor alveolar cell regeneration rather than allow alveolar infiltration by host cells offers the possibility of a novel protective mechanism by DAC in lung transplantation. These regenerated donor alveolar cells do not appear to be quiescent, given their marker profile in comparison to that of alveolar cells from naïve donors. Resident alveolar macrophages, suppressive in nature, are felt to sense, respond to, and be affected by pro-inflammatory stimuli (23). Their capacity for self-replication, temporally associated with the resolution phase of the inflammatory stage of a viral process, may be linked to downregulation of the Wnt-β-catenin pathway (24). In this cascade, β-catenin leads to the transcription of target genes and the self-renewal of hematopoietic and tissue stem cells. Under physiologic conditions, Wnt activates the β-catenin to promote transcription of target genes and self by alveolar macrophages. By contrast, in inflammatory states such as respiratory viral infection, Wnt signaling results in assembly of a “nonconventional” Wnt-b-catenin complex with suppression of alveolar macrophage proliferation. In this setting, DAC has been shown to induce the expression of Wnt antagonists sFRP3 and DKK1, leading to reduced Wnt availability and an increase in alveolar macrophage replication.

### 4.5 Study limitations and conclusion

Our study was not designed to distinguish the relative importance to allograft health of preserving donor resident alveolar macrophage quantities vs. preventing monocyte-derived host macrophage recruitment into the alveoli. Our method of macrophage deletion would be expected to both remove a beneficial effect of donor macrophage preservation and prevent a deleterious consequence of monocyte-derived alveolar macrophages accumulation within the alveoli. Additionally, our manuscript is heavily observational and based on a flow cytometric and histologic approach. Cell depletion and histologic evaluation supporting the flow cytometric findings were also employed. A more extensive evaluation using gain-of-function studies or exploration of the epigenetic factors by which DAC may influence macrophage function was beyond the scope of our study.

Our study confirms that macrophage function contributes to the cascade leading to acute rejection in mouse lung transplant, and that DAC initiated 72 h following lung allograft implantation alters both the pattern of macrophage recruitment into the lung and the pattern of macrophage activation within the lung during acute murine lung allograft rejection. This appears to involve the regeneration of the donor resident alveolar macrophage population. We propose the therapeutic benefit of DAC in acute lung allograft rejection involves an effect on donor and host macrophage populations*.*

## Data Availability

The raw data supporting the conclusions of this article will be made available by the authors, without undue reservation.
