# Supplementary material for: The role of macrophages in the mitigation by decitabine of acute allograft rejection
Source: Front Transplant. 2025 Dec 11;4:1723396. doi: 10.3389/frtra.2025.1723396 (PMC12738378; doi:10.3389/frtra.2025.1723396)
Supplement: Supplementary file 1 [file Datasheet1.docx]

LEGENDS FOR SUPPLEMENTARY FIGURES

**Supplemental Figure 1. List of Antibodies**

**Supplemental Figure 2. Gating Strategy**

**Supplemental Figure 3. Treatment with DAC Alleviates Lung Rejection**

ISHLT A and ISHLT B scoring demonstrating the effect of time, DAC vs DMSO on the observed level of rejection). POD 3 mice were untreated. Mice harvested Day 9 were treated with either vehicle (DMSO, intraperitoneally) or decitabine (DAC, 1 mg/kg, intraperitoneally) on post-transplant days 3, 4, 5, and 8. *N = 4 POD3, 9 DMSO, 11 DAC.*

**Supplemental Figure 4. Contribution of Donor vs Host Cells to Live Cell Suspension**

Box and Whiskers demonstrating host vs donor contribution to total live cells in naïve host and donor lungs, and in allografts of DMSO vs DAC treated hosts. (A) Box and whiskers showing percentage of total live cells in suspension which is comprised of host and donor cells. (B) Box and Whiskers demonstrating ratio of live host vs donor origin immune cells in POD 9 DMSO vs DAC allografts. *Data are presented as mean ± SEM (N = 12 naïve host, 9 POD 9 DMSO, 11 POD 9 DAC, 12 naïve donor). P ≤ 0.05 (*), P ≤ 0.001 (***) and P ≤ 0.0001 (****).*

**Supplemental Figure 5. – Component Host Cells Comprising Live Cell Suspensions (A)** Pie chart showing live host immune cell components as percentage of total live cell suspension in naïve host, POD 9 DMSO allograft, and POD 9 DAC allograft; *N = 10 naïve donor, 9 DMSO day 9, 11 DAC day 9*

**Supplemental Figure 6. – Effect of Clodronate on lung macrophage populations**. Histograms demonstrating the effect of clodronate administration (200 mcl i.v. POD 3, 5, 7 and 20 mcl i.p. POD 4, 6, 8) on lung live interstitial (A) and alveolar (B) macrophage quantities as a percentage of total live cells in suspension. N = 4 per group. P < 0.05 (*).

**Supplemental Figure 7. Effect of DAC on the Phenotype of Host Interstitial Macrophages and Ly6C- Monocyte-Derived Macrophages.** Volcano plot demonstrating the effect of DAC vs. DMSO treatment on the percentage of allograft live host interstitial macrophages and **(B)** live host Ly6C- monocyte-derived macrophages expressing various markers. Effect (x-axis) is presented as Fold Change (Log_2_) in the percentage of live allograft cells expressing a given marker when the host is treated with DAC rather than DMSO. Markers expressed on a higher percentage of cells are depicted more to the right, and increasing statistical significance is depicted by ascending position on the Y axis. The green-shaded region denotes markers with statistically significant differences (p < 0.05, -log_10_ > 1.301). Markers with increased expression in DAC-treated hosts are shown in blue, while those with reduced expression are in red. (N = *9 DMSO, 11 DAC*).

**Supplemental Figure 8. Effect of DAC on the Phenotype of Donor Alveolar Macrophages.** Volcano plot demonstrating the effect of DAC vs. naïve mouse on the percentage of allograft live donor alveolar macrophages expressing various markers. Effect (x-axis) is presented as Fold Change (Log_2_) in the percentage of live allograft cells expressing a given marker when the host is treated with DAC rather than naïve lungs. Markers expressed on a higher percentage of cells are depicted more to the right, and increasing statistical significance is depicted by ascending position on the Y axis. The green-shaded region denotes markers with statistically significant differences (p < 0.05, -log_10_ > 1.301). Markers with increased expression in DAC-treated hosts are shown in blue, while those with reduced expression are in red. (*N = 10 naïve, 11 DAC*).

**Supplemental Figure 9. - Effect of DAC vs Naïve Mice on the Phenotype of Donor Interstitial Macrophages. (A)** Histograms demonstrating the effect of DAC vs. Naïve Mice on the percentage of allograft live host interstitial. Markers reflecting different pathways were grouped together analyzed as such. Data are presented as mean ± SEM. Values were compared with T-tests, with P-values adjusted for multiple testing using the adaptive Benjamini-Krieger-Yekutieli (BKY) false discovery rate (FDR) procedure with a Q value (FDR threshold) of 0.05. (*N = 10 naïve, 11 DAC*). (B) Volcano plots demonstrating the effect of DAC vs. Naïve Mice on the percentage of allograft live host interstitial. Effect (x-axis) is presented as Fold Change (Log_2_) in the percentage of live allograft cells expressing a given marker when the host is treated with DAC rather than DMSO. Markers expressed on a higher percentage of cells are depicted more to the right, and increasing statistical significance is depicted by ascending position on the Y axis. The green-shaded region denotes markers with statistically significant differences (p < 0.05, -log_10_ > 1.301). Markers with increased expression in DAC-treated hosts are shown in blue, while those with reduced expression are in red. (*N = 10 naïve, 11 DAC*).

**Supplemental Figure 10. - DAC Treatment Reduces Host Neutrophils but Increases Host CD103 DC Recruitment into Allograft**

Histogram comparing live (A) host neutrophil and (B) host CD103+ DC counts in POD 9 DAC allografts vs naïve lungs. Data are presented as mean ± SEM . (N = *9 DMSO, 11 DAC*). P ≤ 0.05 (*), P ≤ 0.01 (**), P ≤ 0.001 (***), and P ≤ 0.0001 (****)

**Supplemental Methods**

## *Immunofluorescence staining*

Quadruple immunolabeling for CD4+CD8+CK19+F4/80 was performed at the Oncology Tissue Services Core of Johns Hopkins University School of Medicine on formalin-fixed, paraffin-embedded (FFPE) sections using a Ventana Discovery Ultra autostainer (Roche Diagnostics). After dewaxing and rehydration, epitope retrieval was performed with Ventana Ultra CC1 buffer (Cat. #6414575001, Roche Diagnostics) at 96°C for 64 minutes. Immunostaining was performed sequentially for each marker, with individual rounds consisting of primary antibody incubation at 36°C for 40 minutes, detection using an anti-rabbit HQ detection system (Cat. #7017936001 and #7017812001, Roche Diagnostics), and signal amplification with OPAL fluorophores (Akoya Biosciences) diluted 1:200 in 1X Plus Amplification Diluent (Cat. #FP1498, Akoya Biosciences). For CD8 detection, a rabbit anti-rat linker antibody (1:500; Cat. #AI4001, Vector Labs) was applied at 36°C for 32 minutes before the HQ detection system. The antibodies used were anti-CD4 (1:200; Cat. #ab133616, Abcam) detected with OPAL 570, anti-CD8 (1:125; Cat. #4SM16, Invitrogen) detected with OPAL 690, anti-CK19 (1:1000; Cat. #ab133496, Abcam) detected with OPAL 520, and anti-F4/80 anti‐F4/80 (1:200 dilution; catalog# 70076S, Cell Signaling Technology) detected with OPAL Polaris 780. After each round of staining, primary and secondary antibodies were stripped using Ventana Ultra CC1 buffer at 95°C for 12 minutes, followed by neutralization with Discovery Inhibitor (Cat. #7017944001, Roche Diagnostics). Finally, sections were counterstained with spectral DAPI (Cat. #FP1490, Akoya Biosciences) and mounted with Prolong Gold (Cat. #P36930, ThermoFisher Scientific). Slides were viewed and scanned using the Olympus IX83 Inverted Microscope FISHscope and the Olympus CellSens software. Images were analyzed using ImageJ.
