# Supplementary figures and images for: The role of macrophages in the mitigation by decitabine of acute allograft rejection
Source: Front Transplant. 2025 Dec 11;4:1723396. doi: 10.3389/frtra.2025.1723396 (PMC12738378; doi:10.3389/frtra.2025.1723396)

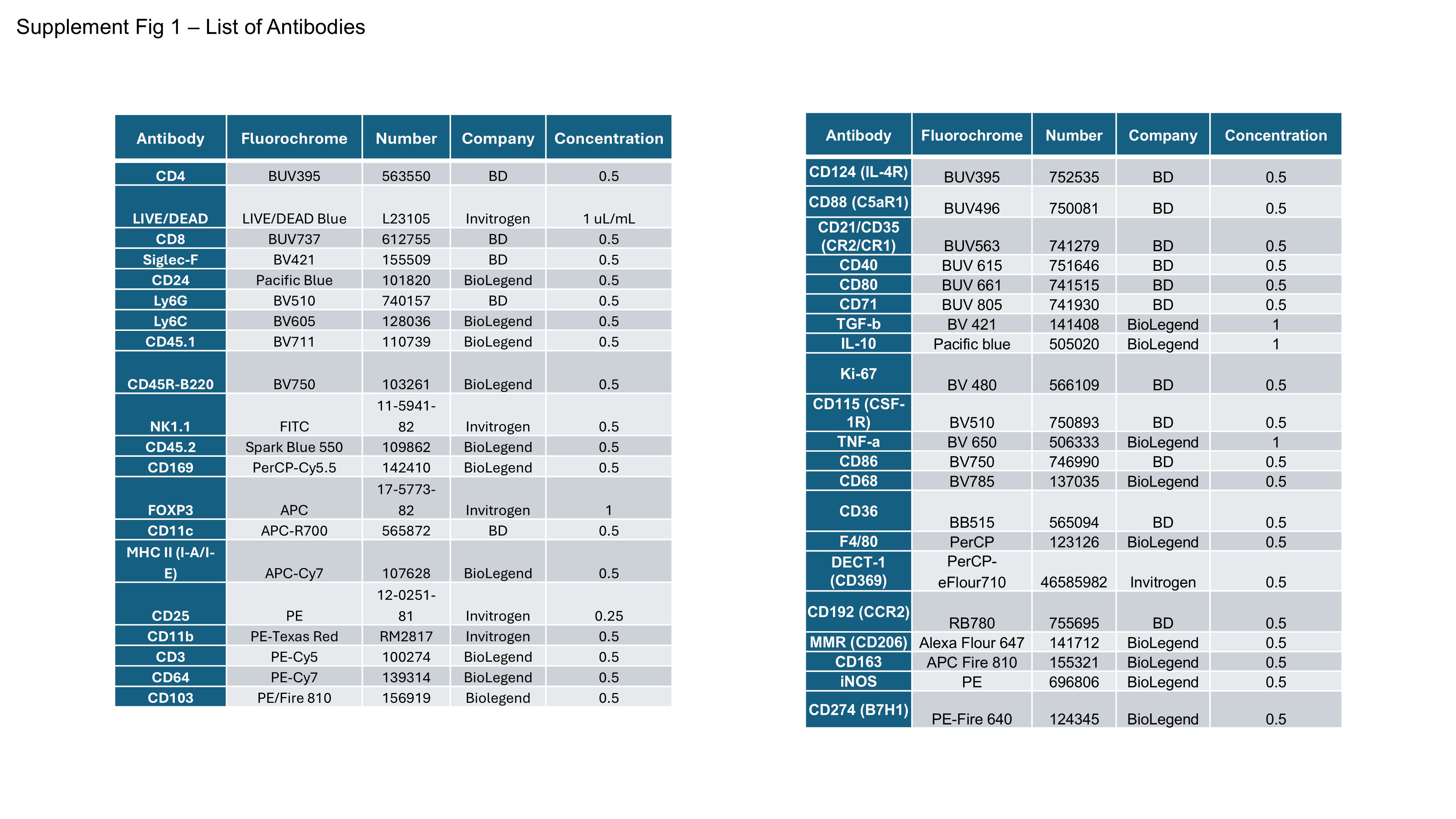

Supplement: Supplementary file 2 [file Image1.jpeg]

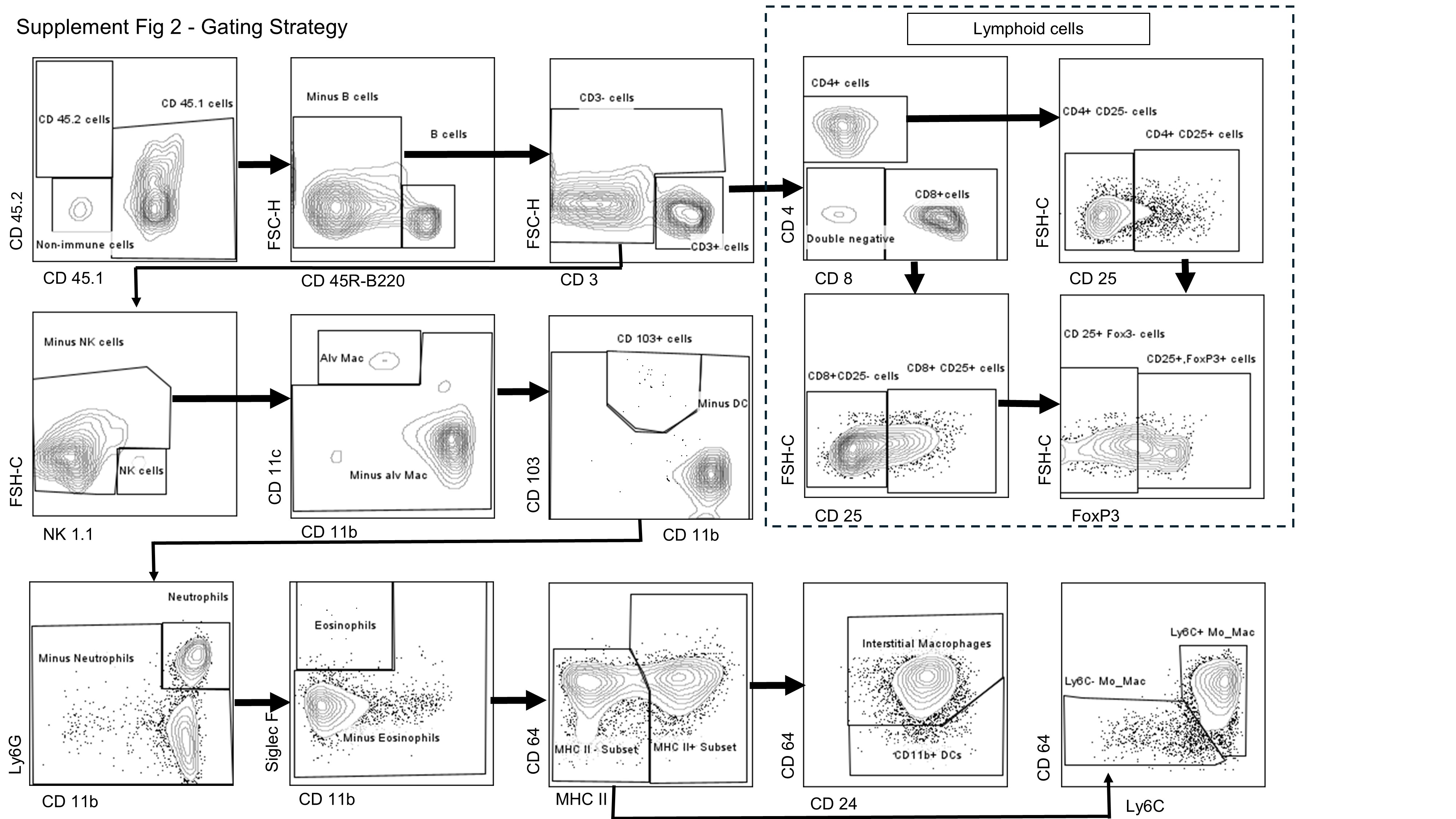

Supplement: Supplementary file 3 [file Image2.jpeg]

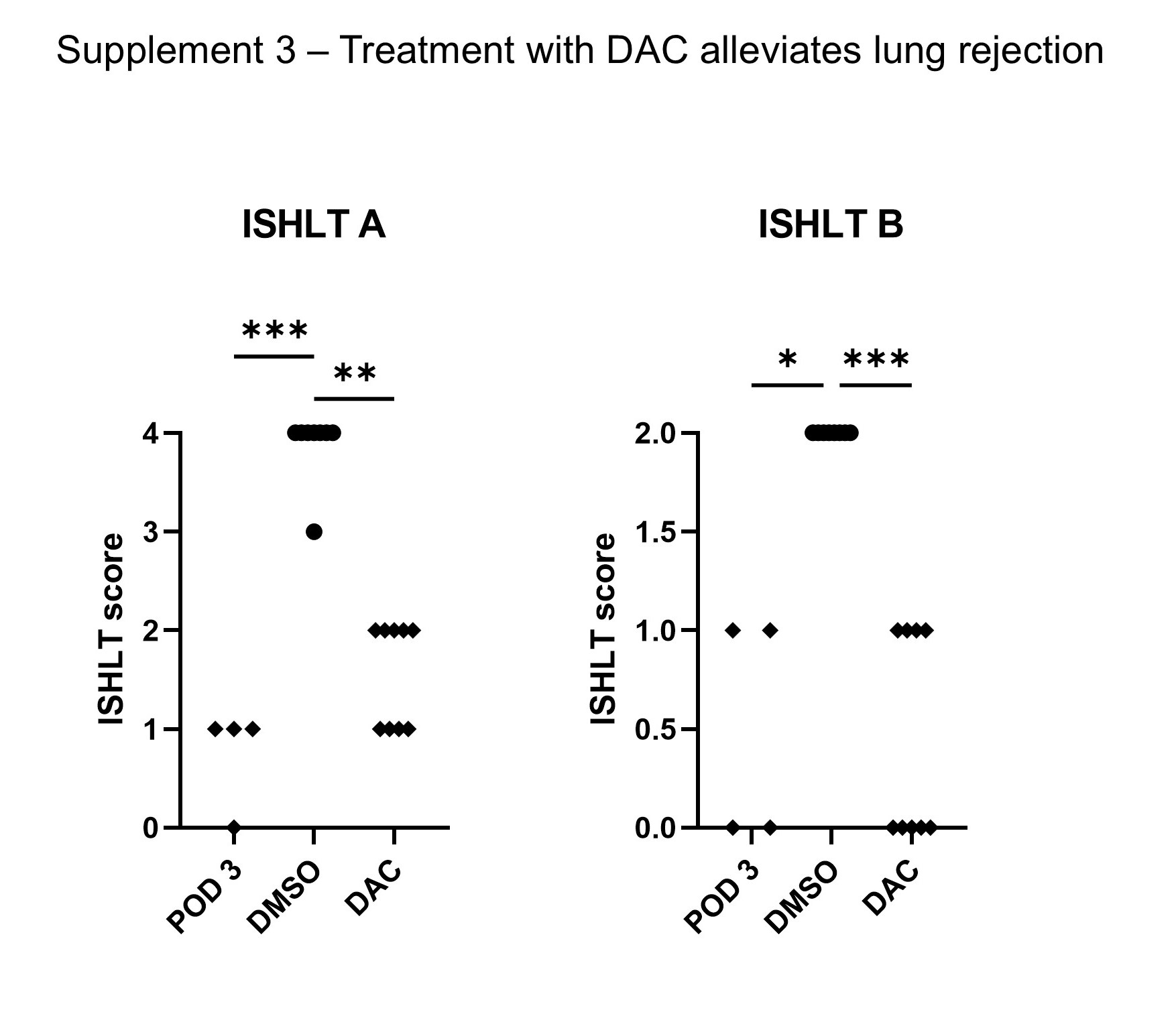

Supplement: Supplementary file 4 [file Image3.jpeg]

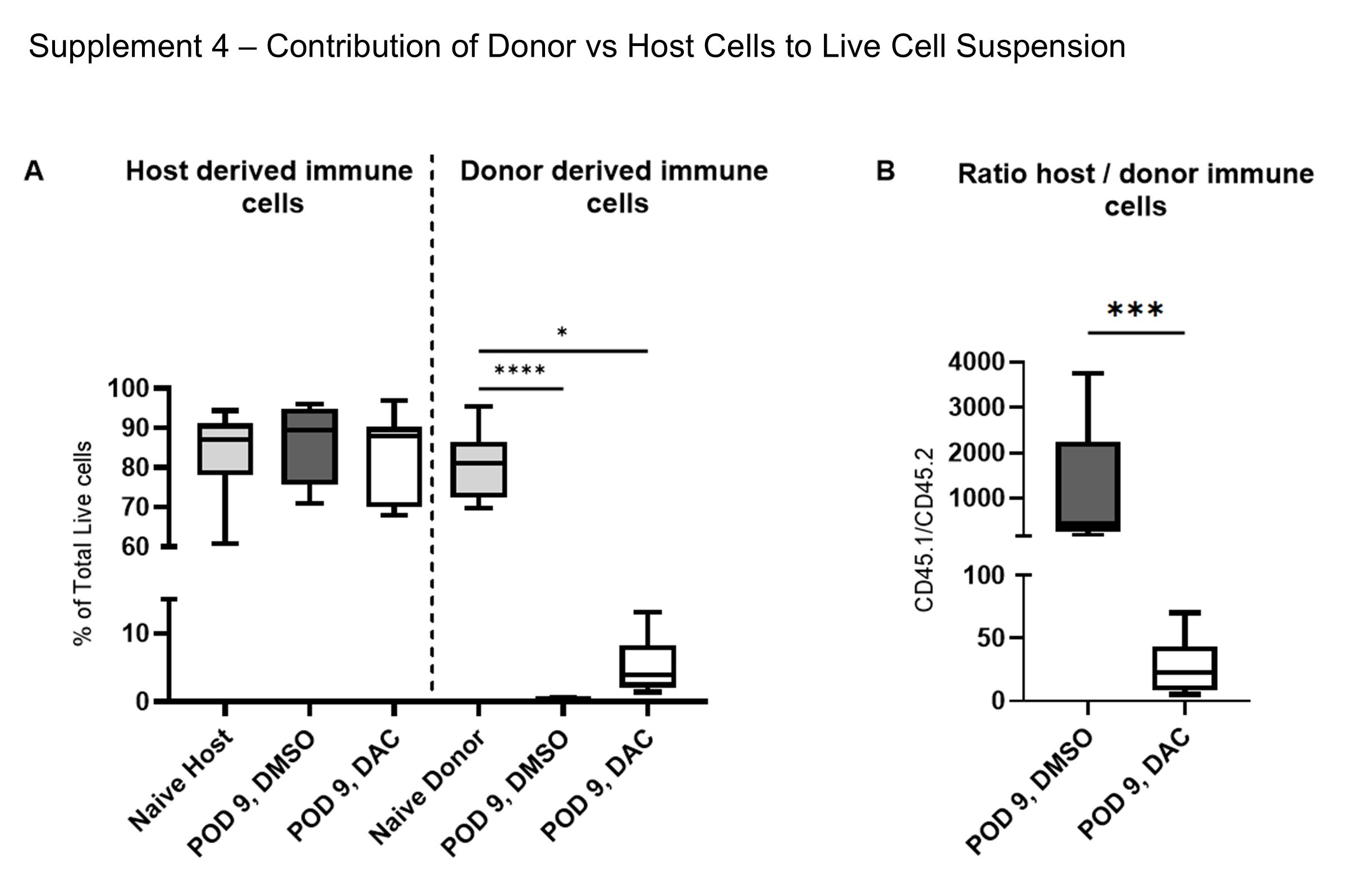

Supplement: Supplementary file 5 [file Image4.jpeg]

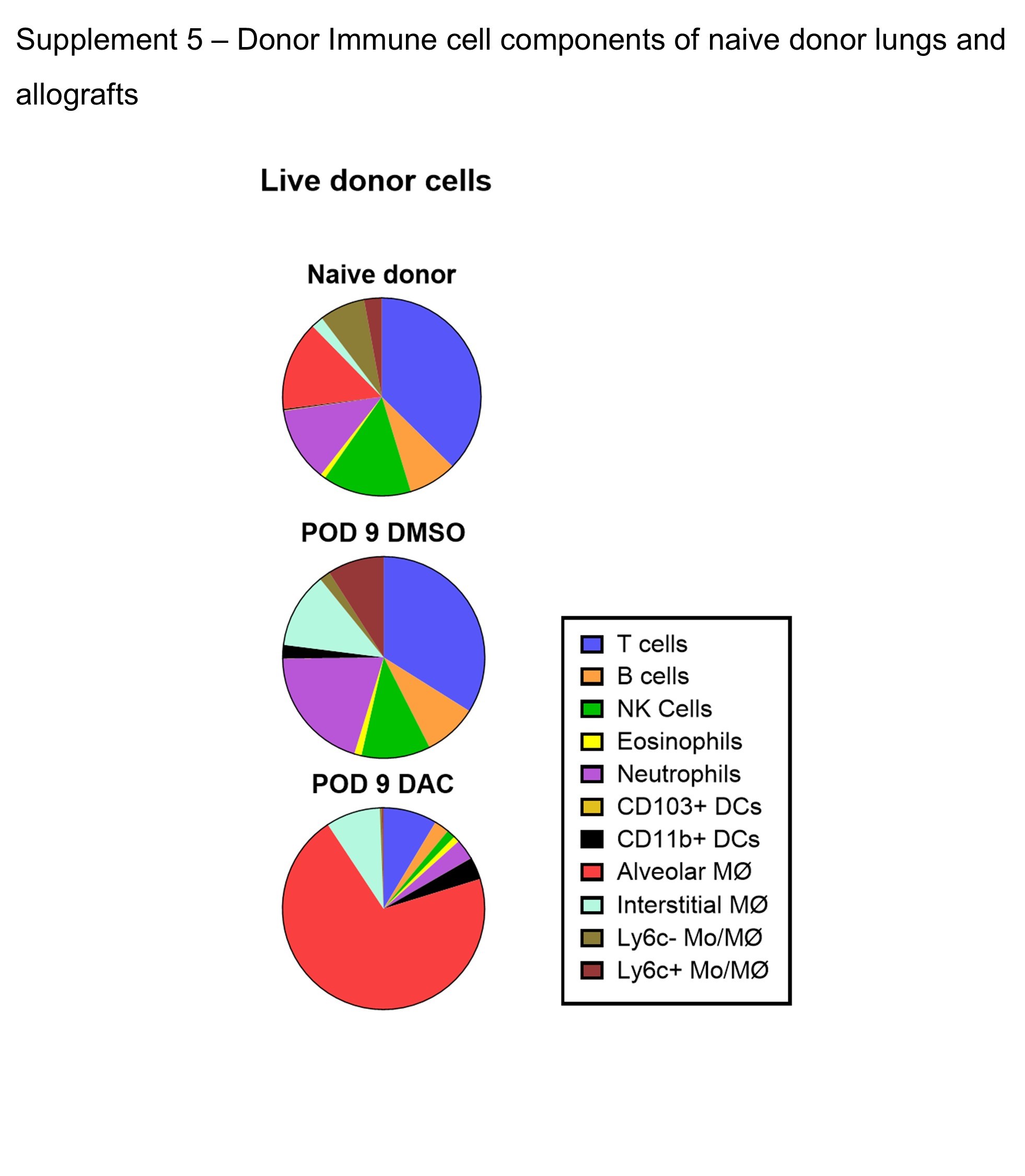

Supplement: Supplementary file 6 [file Image5.jpeg]

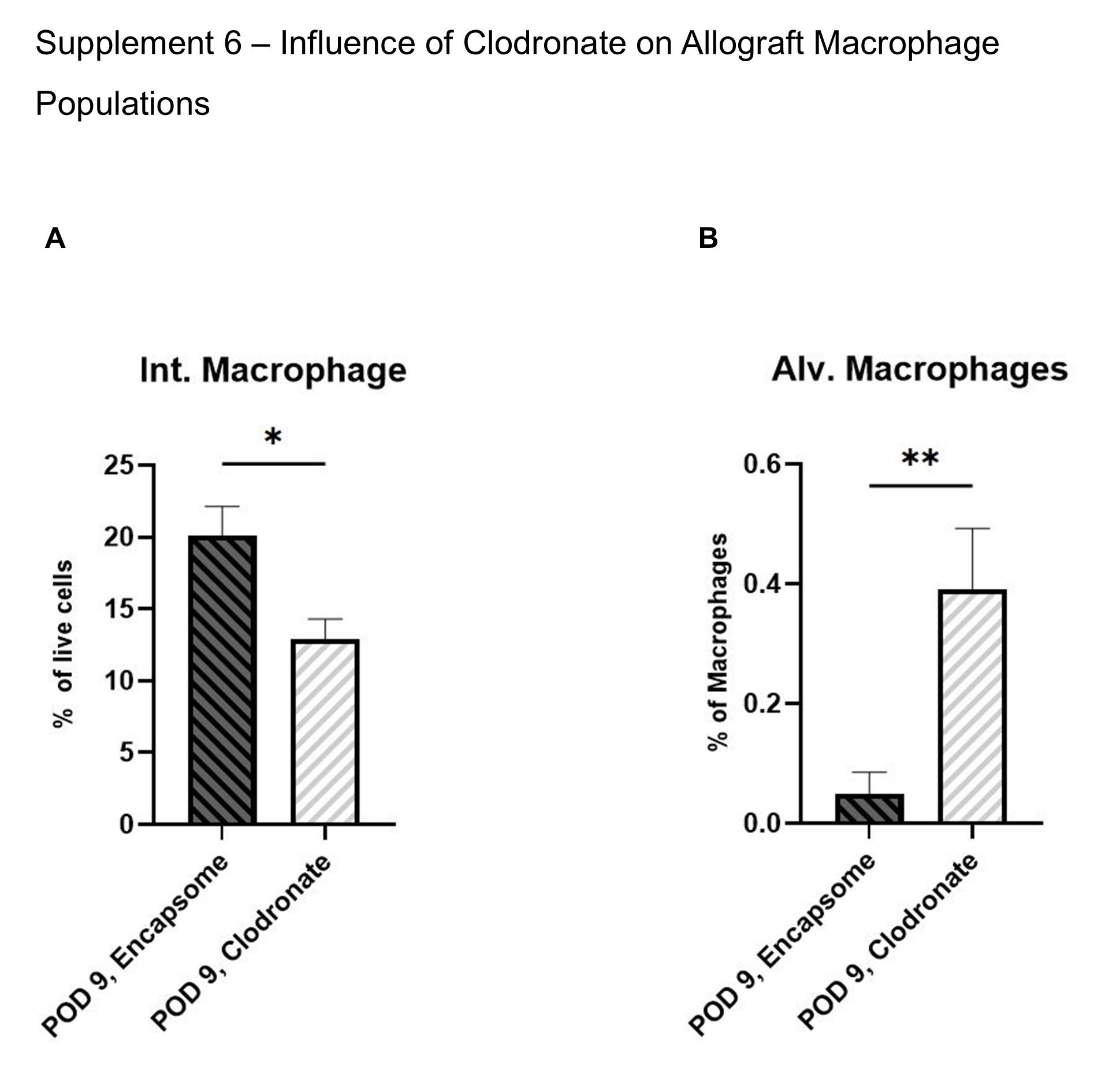

Supplement: Supplementary file 7 [file Image6.jpeg]

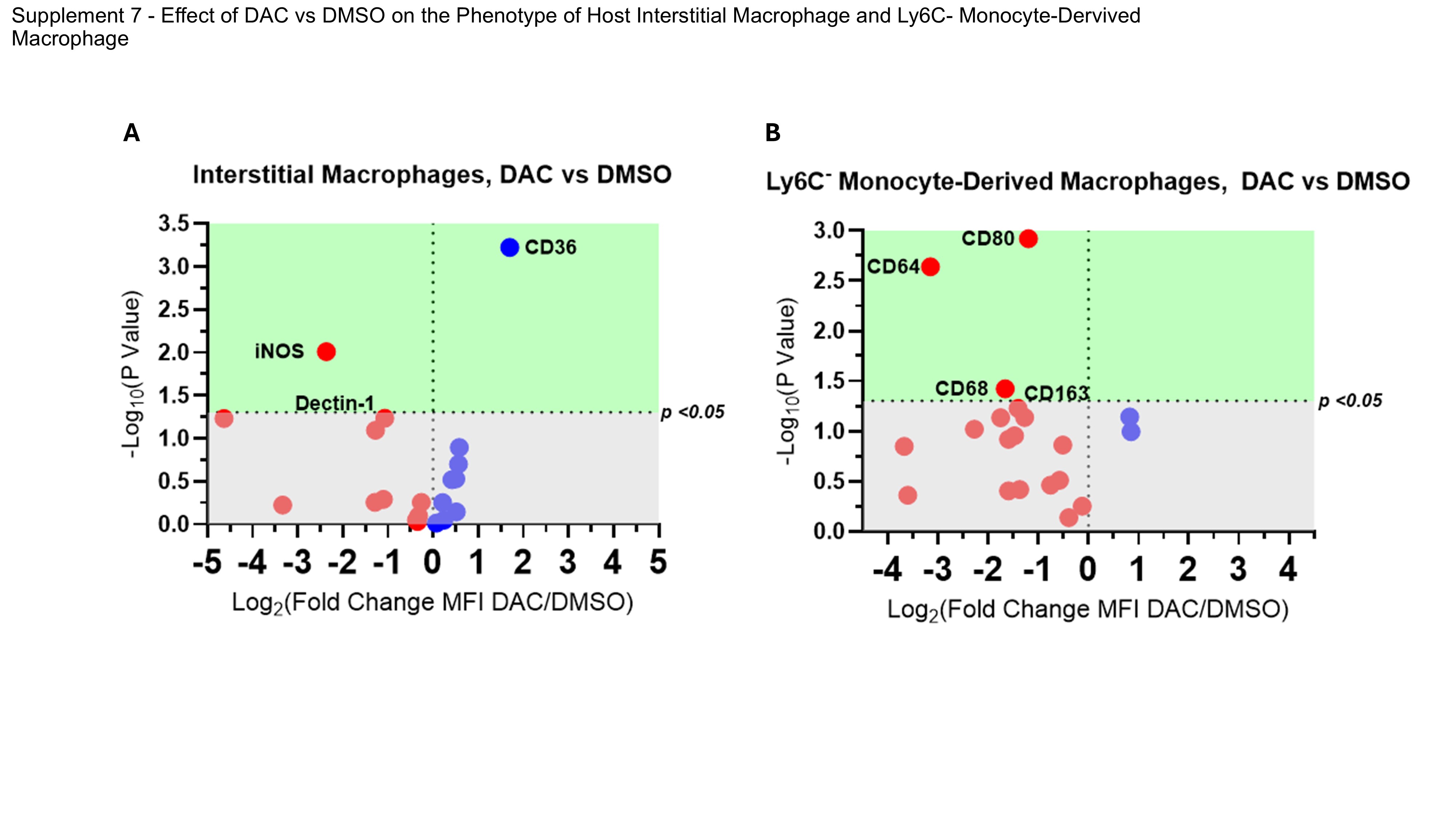

Supplement: Supplementary file 8 [file Image7.jpeg]

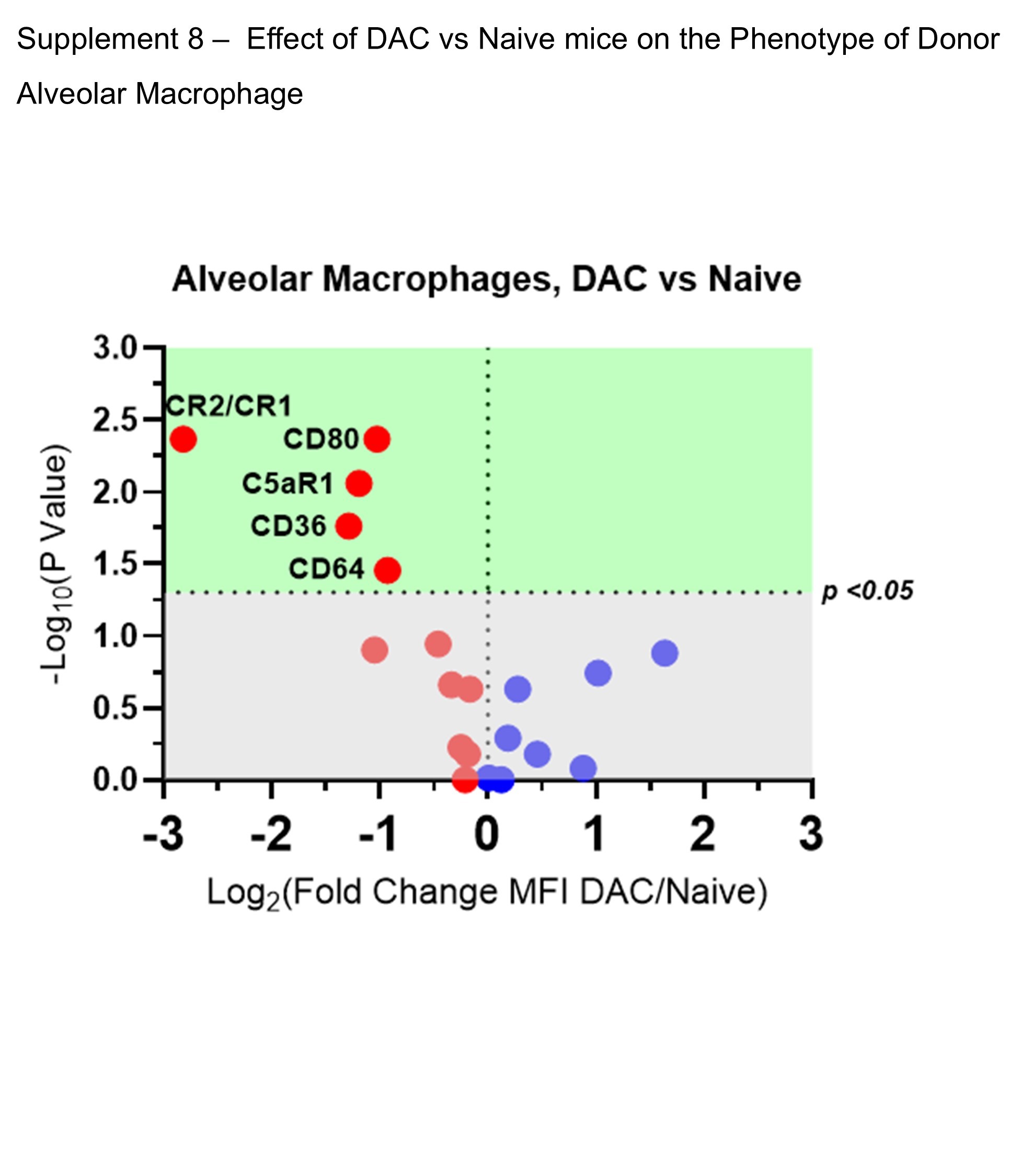

Supplement: Supplementary file 9 [file Image8.jpeg]

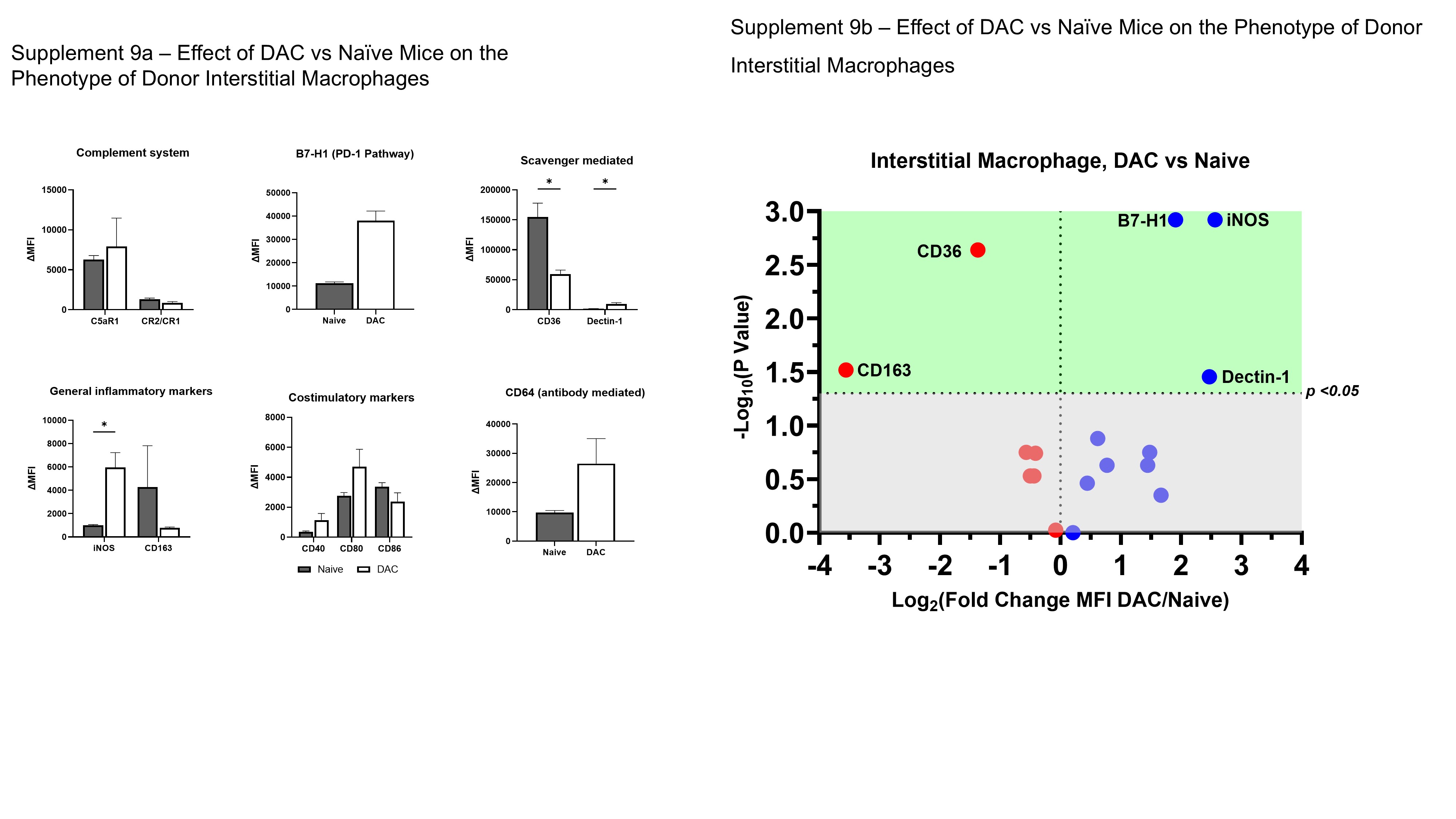

Supplement: Supplementary file 10 [file Image9.jpeg]

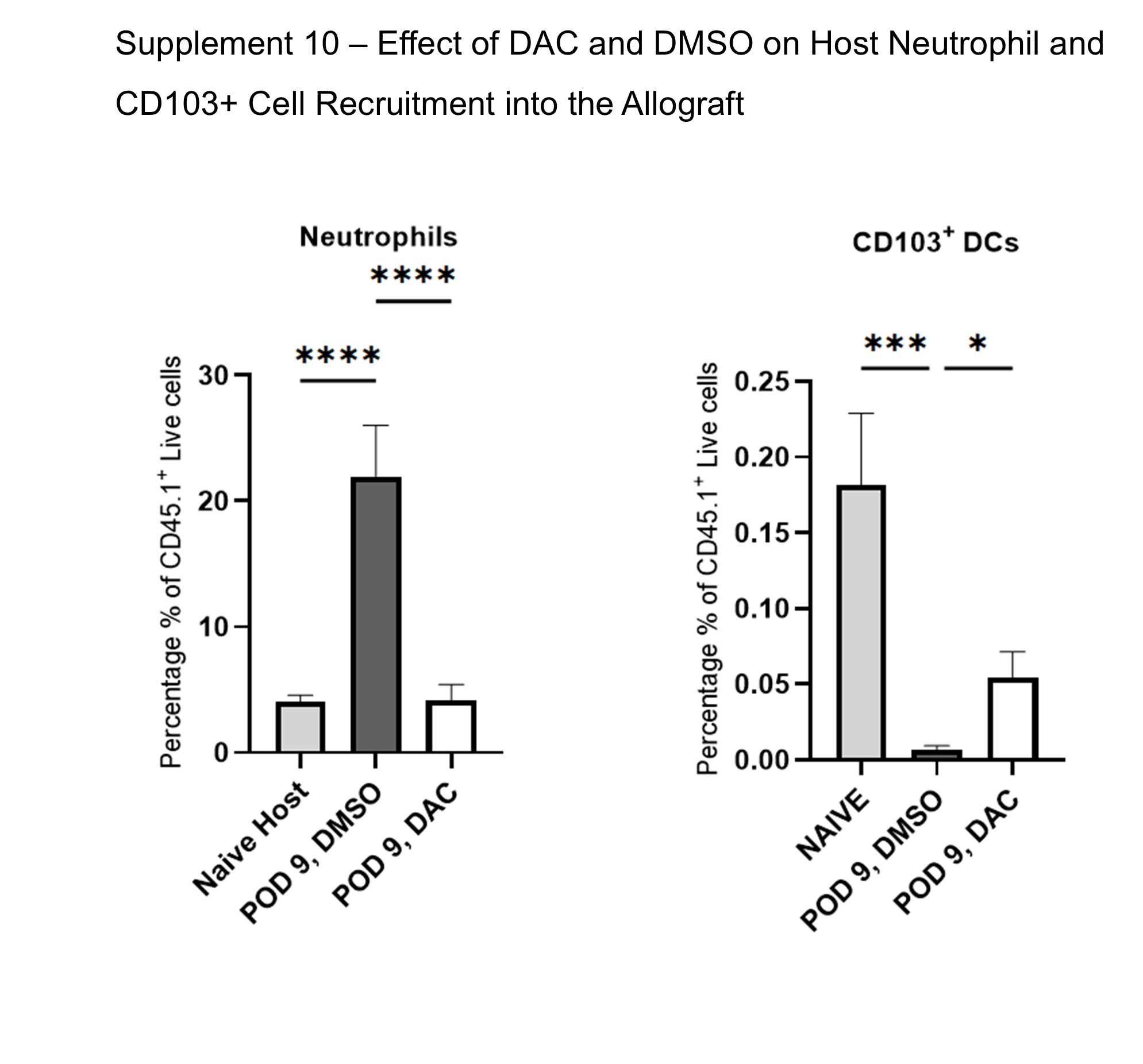

Supplement: Supplementary file 11 [file Image10.jpeg]
